# DHODH is an independent prognostic marker and potent therapeutic target in neuroblastoma

**DOI:** 10.1172/jci.insight.153836

**Published:** 2022-09-08

**Authors:** Thale Kristin Olsen, Cecilia Dyberg, Bethel Tesfai Embaie, Adele Alchahin, Jelena Milosevic, Jane Ding, Jörg Otte, Conny Tümmler, Ida Hed Myrberg, Ellen M. Westerhout, Jan Koster, Rogier Versteeg, Han-Fei Ding, Per Kogner, John Inge Johnsen, David B. Sykes, Ninib Baryawno

**Affiliations:** 1 Division of Pediatric Oncology and Pediatric Surgery, Department of Women’s and Children’s Health, Karolinska Institutet, Stockholm, Sweden.; 2 Department of Immunology, Genetics and Pathology, Uppsala University, Uppsala, Sweden.; 3 Center for Regenerative Medicine, Massachusetts General Hospital, Boston, Massachusetts, USA.; 4 Division of Molecular and Cellular Pathology, Department of Pathology, Heersink School of Medicine, the University of Alabama at Birmingham, Birmingham, Alabama, USA.; 5 Department of Oncogenomics, Amsterdam UMC, University of Amsterdam, Amsterdam, Netherlands.; 6 Harvard Stem Cell Institute, Cambridge, Massachusetts, USA.

**Keywords:** Oncology, Therapeutics, Cancer

## Abstract

Despite intensive therapy, children with high-risk neuroblastoma are at risk of treatment failure. We applied a multiomic system approach to evaluate metabolic vulnerabilities in human neuroblastoma. We combined metabolomics, CRISPR screening, and transcriptomic data across more than 700 solid tumor cell lines and identified dihydroorotate dehydrogenase (DHODH), a critical enzyme in pyrimidine synthesis, as a potential treatment target. Of note, DHODH inhibition is currently under clinical investigation in patients with hematologic malignancies. In neuroblastoma, *DHODH* expression was identified as an independent risk factor for aggressive disease, and high *DHODH* levels correlated to worse overall and event-free survival. A subset of tumors with the highest *DHODH* expression was associated with a dismal prognosis, with a 5-year survival of less than 10%. In xenograft and transgenic neuroblastoma mouse models treated with the DHODH inhibitor brequinar, tumor growth was dramatically reduced, and survival was extended. Furthermore, brequinar treatment was shown to reduce the expression of MYC targets in 3 neuroblastoma models in vivo. A combination of brequinar and temozolomide was curative in the majority of transgenic TH-*MYCN* neuroblastoma mice, indicating a highly active clinical combination therapy. Overall, DHODH inhibition combined with temozolomide has therapeutic potential in neuroblastoma, and we propose this combination for clinical testing.

## Graphical abstract

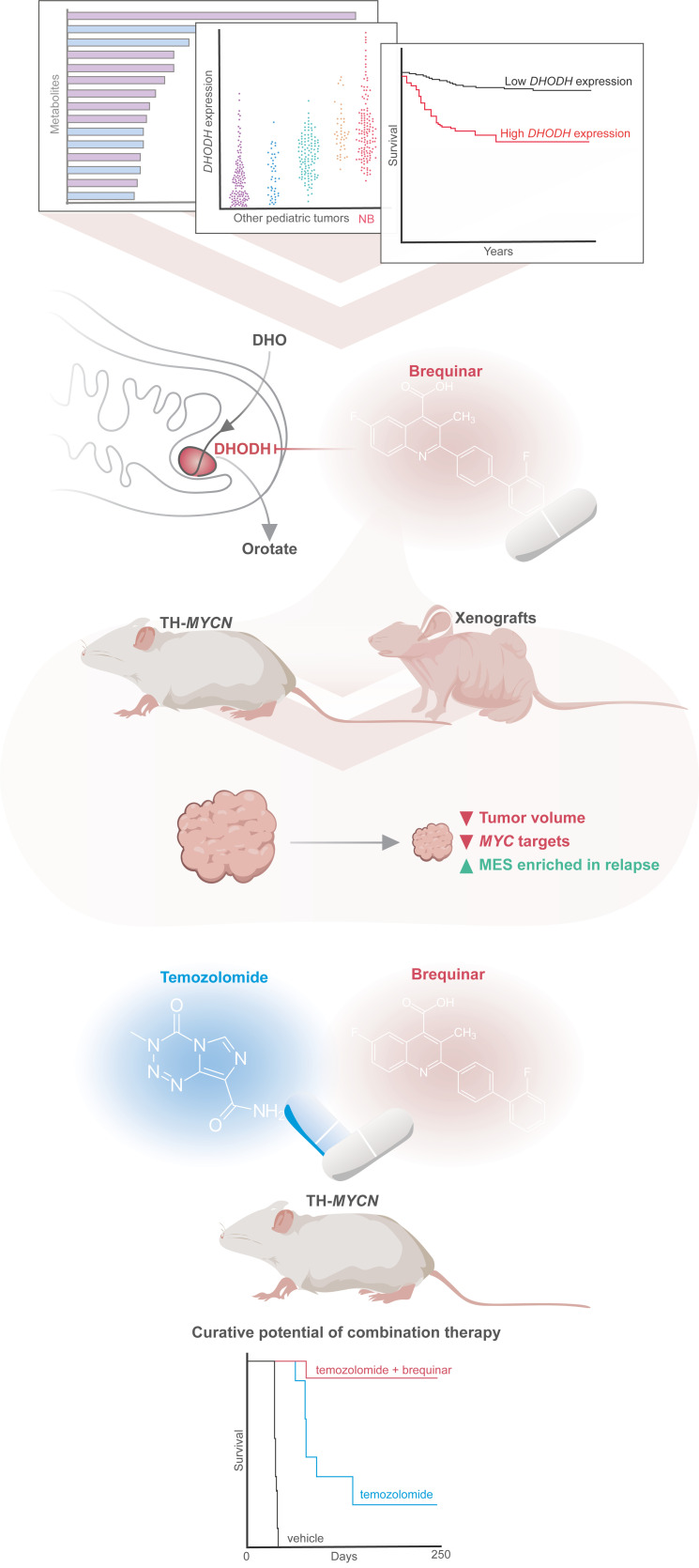

## Introduction

Neuroblastoma is an embryonal childhood tumor that manifests in the tissue of the adrenal medulla or paraspinal or other sympathetic ganglia and accounts for about 7%–10% of all childhood cancers (1–3). Despite intensive multimodal therapy, survival in the high-risk group of patients with neuroblastoma remains less than 50% (1). The N-myc proto-oncogene (*MYCN*) is frequently amplified in neuroblastoma (around 20% of cases) and is one of the defining hallmarks of high-risk disease (1, 4). MYCN is a transcription factor whose target genes are widely involved in metabolism, apoptosis, and cell growth (5–7), and its dysregulation in neuroblastoma leads to increased proliferation, decreased apoptosis, and differentiation arrest (8, 9). *MYCN* amplification characterizes a subset of very aggressive tumors and correlates with poor prognosis. Moreover, the closely related c-myc transcription factor (*MYC*) has been established as a potent oncogene in an additional subset of neuroblastoma (approximately 10% of cases) (10). Oncogenic *MYC* is known to orchestrate a profound rewiring of the metabolic processes in cancer cells. By driving the expression of metabolic genes, *MYC* increases production of ATP and other key cellular building blocks, which enable cell proliferation (11, 12). MYCN is also known to induce several metabolic alterations, such as increased glycolysis, oxidative phosphorylation, and glutamine metabolism (13–15). Unconstrained growth in *MYC-* and *MYCN-*driven cancers is thus dependent on metabolic pathways, which may serve as novel targets for cancer therapy.

Genome-wide epigenetic and transcriptomic studies in neuroblastoma cell lines have demonstrated at least 2 malignant neuroblastoma cell types, namely the mesenchymal (MES) and adrenergic (ADRN) cells, which to a large extent are controlled by networks of super-enhancers (16, 17). In a recent study of the super-enhancer landscapes in primary neuroblastoma patient samples, the presence of these 2 states was confirmed, and the ADRN phenotype was found to comprise 3 distinct subtypes: *MYCN-*amplified, non–*MYCN*-amplified high-risk, and non–*MYCN*-amplified low-risk signatures (18). The MES phenotype was significantly enriched in relapsed tumors (18) and has been linked to chemotherapy resistance (17).

Cancer cells activate the purine and pyrimidine biosynthesis pathways to meet the nucleotide demands of rapidly proliferating cells (19). There is increasing evidence that dihydroorotate dehydrogenase (DHODH), an essential enzyme for de novo pyrimidine synthesis, is a promising therapeutic target in various cancer types, including melanoma, acute myeloid leukemia, glioblastoma, lung cancer, pancreatic cancer, and prostate cancer (20–25). We recently demonstrated that the DHODH inhibitor brequinar is an effective differentiation therapy in myeloid leukemia, demonstrating prolonged survival in mouse and PDX models due to differentiation of leukemic stem cells into mature myeloid cells (26). Multiple DHODH inhibitors are currently under investigation in human cancer clinical trials (ClinicalTrials.gov, identifiers NCT03760666, NCT03709446, NCT02509052). It was recently demonstrated that DHODH inhibition may suppress tumor growth in vivo by inducing ferroptosis (27). However, the mechanisms by which DHODH inhibition induces the differentiation of malignant cells still remain largely unknown.

In this study we applied a pancancer, multiomic approach to elucidate metabolic dependencies in cancer and identified critical roles of DHODH, both as a prognostic marker and as a mediator of tumor cell survival in neuroblastoma. We report that the therapeutic combination of DHODH inhibition and the standard-of-care chemotherapeutic temozolomide has curative potential in a transgenic neuroblastoma mouse model and may be a promising candidate for the treatment of high-risk neuroblastoma.

## Results

### Neuroblastoma cells accumulate nucleotide metabolites and express high levels of DHODH.

**Figure 1:**
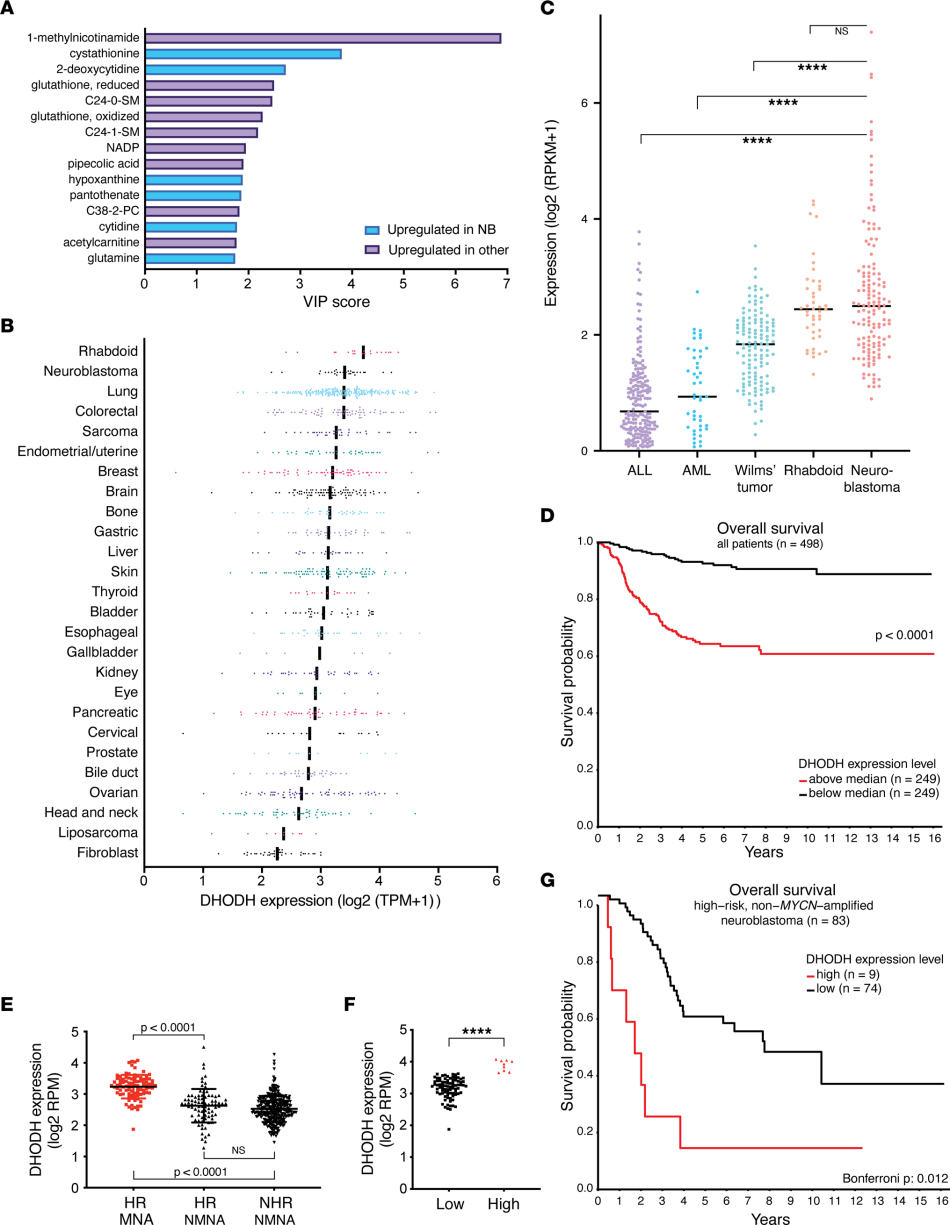
*DHODH* is highly expressed in neuroblastoma, and expression correlates with disease stage, prognosis, and survival. (**A**) Top 15 accumulated metabolites in neuroblastoma cell lines compared with other solid tumor cell lines of the Cancer Cell Line Encyclopedia (CCLE) metabolomics data set. Metabolite enrichment in neuroblastoma is quantified as variable importance in projection scores generated through partial least-squares discriminant analysis. (**B**) *DHODH* expression in solid tumor cell lines of the CCLE data set, grouped by primary disease. Black lines represent median values. (**C**) *DHODH* expression in primary pediatric tumor samples of the TARGET data set. Black lines represent median values. ****: *P* < 0.0001 (as evaluated by 1-way ANOVA with multiple comparisons). ALL, acute lymphatic leukemia; AML, acute myeloid leukemia. (**D**) Overall survival in 498 patients with neuroblastoma (SEQC-498 data set), separated by median *DHODH* expression. Red, *DHODH* above median (high); black, *DHODH* below median (low). Groups are compared using log-rank test. (**E**) *DHODH* expression in 498 neuroblastoma samples (SEQC-498 data set). *DHODH* expression is significantly higher in the *MYCN-*amplified tumors as evaluated by 1-way ANOVA with multiple comparisons. TPM, transcripts per million; RPKM, reads per kilobase million; RPM; reads per million; HR, high-risk; MNA, *MYCN-*amplified; NHR, non–high-risk; NMNA, non–*MYCN-*amplified. (**F**) *DHODH* expression levels in high-risk, non–*MYCN-*amplified tumors. High/low groups are identified using the KaplanScan feature of the R2 database (http://r2.amc.nl). Groups were compared using Student’s 2-tailed *t* test. (**G**) Kaplan-Meier curve showing the survival of the groups in **F**. Note that the “highest DHODH group” (*n* = 9) is associated with a particularly poor overall survival. Bonferroni method is used to correct for multiple testing.

In order to explore metabolic dependencies and characteristics in neuroblastoma, we used the Cancer Cell Line Encyclopedia (CCLE) metabolome data set. Comparing a panel of neuroblastoma cell lines (*n* = 17) to a wide range of solid tumor cell lines (*n* = 746) using partial least-squares discriminant analysis, we identified several metabolites that were expressed at high levels in neuroblastoma compared with other solid cancers (Figure 1A and Supplemental Figure 1A; supplemental material available online with this article; https://doi.org/10.1172/jci.insight.153836DS1). These include the pyrimidines 2-deoxycytidine and cytidine, the purine derivative hypoxanthine, and the versatile nutrient glutamine, which provides nitrogen for nucleotide synthesis (28). Due to the accumulation of 2-deoxycytidine and cytidine, we hypothesized that pyrimidine metabolism may play an important role in neuroblastoma and therefore curated a list of 16 genes encoding enzymes related to pyrimidine synthesis and salvage (Supplemental Table 1). Next, we studied the vulnerability to CRISPR-mediated genetic perturbation of these enzymes by assessing CERES scores for a panel of 990 cancer cell lines, using the DepMap resource from the Broad Institute (https://depmap.org). A lower CERES score indicates that the cells are more likely to be dependent on that particular gene. Genes encoding the enzymes carbamoyl-phosphate synthetase 2 (CAD), DHODH, and uridine monophosphate synthetase (UMPS), which catalyze subsequent steps of de novo pyrimidine synthesis, were found to correlate strongly to each other in the DepMap data set, with Pearson correlation scores more than 0.7 (Supplemental Table 1). Cancer cell lines that are highly dependent on 1 enzyme of de novo pyrimidine synthesis were also highly dependent on the other 2, suggesting that such cancers were “addicted” to UMP production and pyrimidines. This strong intercorrelation between CAD, DHODH, and UMPS was also evident in the subset of neuroblastoma cell lines (Supplemental Figure 1B).

In comparison with other solid tumor cell lines (Figure 1B) and primary pediatric tumor samples (Figure 1C), *DHODH* was expressed at high levels in neuroblastoma and rhabdoid tumors. Consequently, we next sought to explore the clinical relevance of DHODH in neuroblastoma.

### DHODH is an independent unfavorable prognostic marker in neuroblastoma.

We next evaluated 2 independent cohorts to investigate the prognostic implications of *DHODH* expression in human neuroblastoma. High *DHODH* expression was significantly correlated with poor survival when combining all stages of the disease (Figure 1D and Supplemental Figure 1D). Interestingly, the highest levels of *DHODH* expression were observed in high-risk, *MYCN*-amplified tumors (Figure 1E), and *DHODH* expression increased with International Neuroblastoma Staging System (INSS) stage (Supplemental Figure 1C). Cox regression analysis of 2 independent cohorts, with adjustment for age, INSS stage, risk group, and *MYCN* status, showed that increased *DHODH* expression was an independent risk factor significantly correlated with unfavorable overall survival (Supplemental Table 2). Notably, even within the high-risk and non–high-risk groups, high *DHODH* expression (above median) correlated significantly with worse survival in non–*MYCN*-amplified cases (Supplemental Figure 1, F and G). Importantly, a subset of high-risk, non–*MYCN*-amplified cases with very high *DHODH* expression (Figure 1F) demonstrated dismal prognosis: a “highest risk group” (Figure 1G). Taken together, high *DHODH* expression is significantly correlated with adverse outcomes in patients with neuroblastoma.

### DHODH inhibition is an effective therapy in preclinical models of neuroblastoma.

The effect of DHODH inhibition on tumor cell growth was evaluated in a panel of neuroblastoma cell lines and a nonmalignant fibroblast cell line. We used the DHODH inhibitor brequinar, which has demonstrated promising effects in both human and murine AML models (26) and is currently under investigation in human clinical trials (ClinicalTrials.gov identifier: NCT03760666). Brequinar demonstrated suppression of tumor cell growth across a panel of neuroblastoma cell lines, with IC_50_ values in the low nanomolar range (Supplemental Figure 2, A and B, and Supplemental Table 3). One notable exception was the SH-EP cell line, which was highly resistant to brequinar treatment.

**Figure 2:**
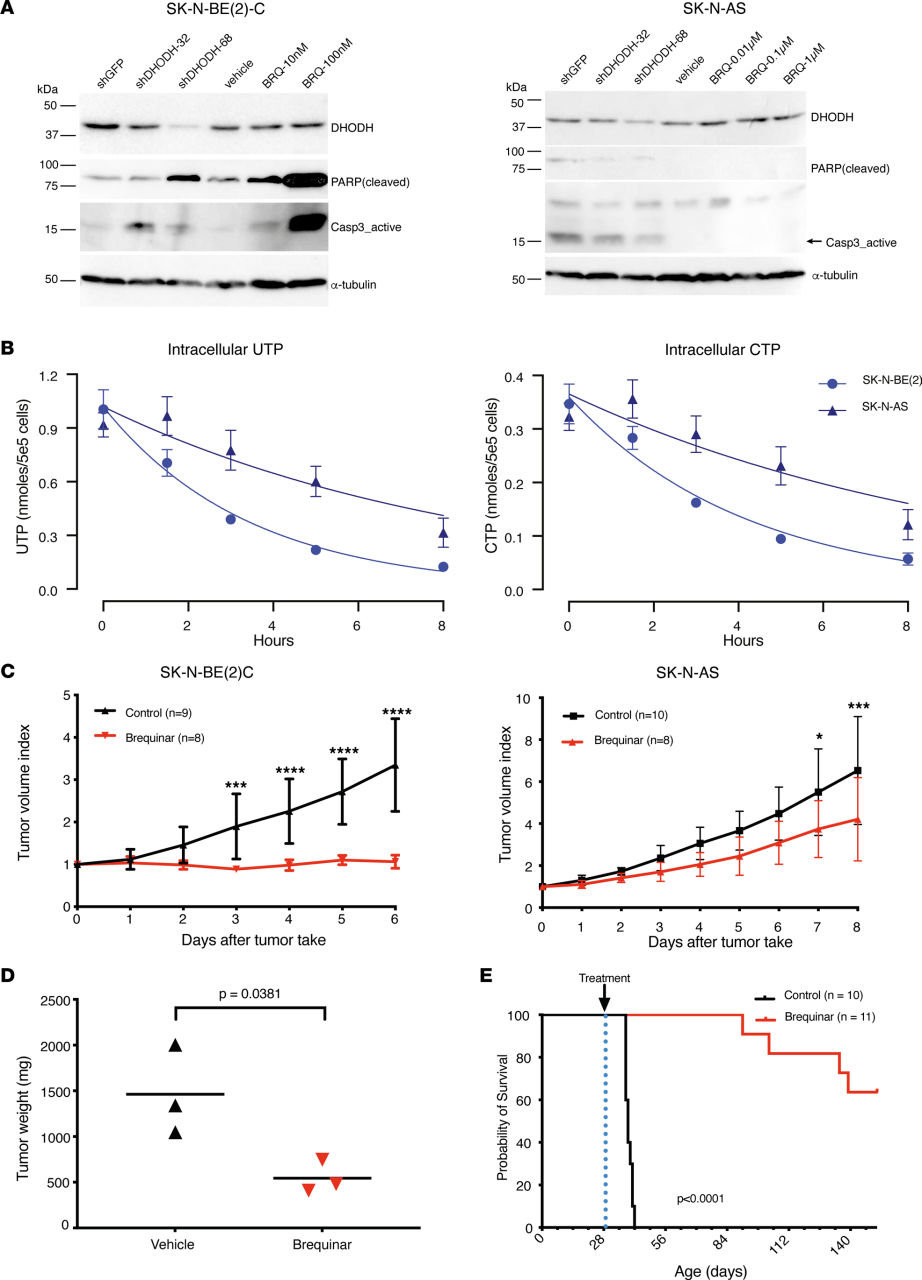
DHODH inhibition reduces neuroblastoma growth in vivo. (**A**) Genetic and pharmacological inhibition of DHODH induces apoptosis in SK-N-BE(2)C but not SK-N-AS cells in vitro. Western blots demonstrate protein expression of DHODH as well as cleaved PARP and activated caspase-3 (apoptosis markers) after shRNA DHODH knockdown and brequinar treatment. (**B**) Inhibition of DHODH decreases the pool of pyrimidines in neuroblastoma cell lines. UTP (left panel) and CTP (right panel) decay in SK-N-BE(2) and SK-N-AS cells following treatment with brequinar (1 μM). (**C**) Tumor volumes of SK-N-BE(2) and SK-N-AS NMRI *nu/nu* xenografts treated with brequinar 50 mg/kg intraperitoneally every 3 days. Tumor volume index at a given day is calculated as the tumor volume relative to the volume at the time of inclusion. Error bars and symbols indicate mean with SD. For each time point, groups are compared with Student’s 2-tailed *t* test adjusted with Holm-Šidák method. *: *P* < 0.05; ***: *P* < 0.001; ****: *P* < 0.0001. (**D**) Tumor weight in homozygous TH-*MYCN* mice 72 hours after receiving 1 dose of either brequinar or vehicle. Error bars show mean with SD. Groups are compared using Student’s 2-sided *t* test. (**E**) Kaplan-Meier curve of homozygous TH-*MYCN* mice treated with brequinar 50 mg/kg intraperitoneally. Treatment started at 32 days of age (marked with dotted line) and continued for 120 days when possible. Groups are compared using log-rank test.

We sought to evaluate the cellular effects of DHODH inhibition in vitro and performed short hairpin RNA (shRNA) knockdown of *DHODH* in the *MYCN-*amplified SK-N-BE(2)C and the non–*MYCN-*amplified SK-N-AS cell lines (Figure 2A). *DHODH* knockdown was confirmed at protein level by Western blot analysis. In SK-N-BE(2)C cells, DHODH inhibition by both genetic perturbation (shRNA) as well as brequinar treatment induced apoptosis as evaluated by PARP cleavage and caspase-3 activation (Figure 2A). Similar observations were made in vivo, where brequinar treatment induced caspase-3 activation in SK-N-BE(2)C xenograft tumors (Supplemental Figure 2H). In gene set enrichment analyses (GSEAs) of RNA-Seq data from SK-N-AS and SK-N-BE(2)C xenograft tumors, we observed upregulation of apoptosis-related genes in tumors from both cell lines after 1 dose of brequinar (Supplemental Table 4). In contrast, SK-N-AS cells did not show any protein-level evidence of apoptosis after genetic or pharmacological inhibition of DHODH (Figure 2A).

To confirm the on-target effect of pyrimidine synthesis in neuroblastoma cell lines, SK-N-BE(2)C and SK-N-AS cells were treated with brequinar, and samples were processed for quantitation of intracellular nucleotides by high-performance liquid chromatography (HPLC) (Figure 2B). Inhibition of DHODH by brequinar resulted in a more rapid depletion of intracellular nucleotides in the SK-N-BE(2)C cells, suggesting an increased dependence on de novo synthesis and an inability to adequately salvage extracellular nucleotides to meet cellular demands. Notably, the intracellular purine pools were spared, speaking to the on-target specificity of brequinar (Supplemental Figure 2C). To control for the potential confounder of cell proliferation rate, a carboxyfluorescein succinimidyl ester proliferation assay was performed, which demonstrated a highly similar doubling time between the 2 cell lines (Supplemental Figure 2D).

Next, we evaluated the cell cycle activity after shRNA *DHODH* knockdown by flow cytometric analysis (Supplemental Figure 2E). In SK-N-AS, we observed G1 arrest, with a reduction of cells in S phase. In SK-N-BE(2)C, on the other hand, we observed an S phase arrest with an increase of cells in S phase.

To evaluate the therapeutic potential of DHODH inhibition in vivo, mice bearing SK-N-BE(2)C or SK-N-AS xenograft tumors were treated with brequinar. These cell lines were selected based on their *MYCN* amplification status [SK-N-BE(2)C: *MYCN*-amplified; SK-N-AS: non–*MYCN*-amplified] and the difference in sensitivity to DHODH inhibition in vitro (Supplemental Figure 2A). Comparable to our in vitro observations, brequinar treatment dramatically suppressed tumor growth in SK-N-BE(2)C xenografts, with a less pronounced effect in the SK-N-AS xenograft model (Figure 2C).

Since prodifferentiating effects of brequinar have been demonstrated in AML (26, 29, 30), we investigated whether the neuroblastoma xenografts still had the potential to relapse following cessation of brequinar therapy. After treatment cessation at day 18, SK-N-BE(2)C xenografts quickly recurred, indicating that the malignant cells were not completely eradicated or terminally differentiated (Supplemental Figure 2F).

To evaluate the therapeutic potential of brequinar in an immunocompetent in vivo model, we treated homozygous transgenic TH-*MYCN* neuroblastoma mice (31) with brequinar once abdominal tumors were palpable. A single dose resulted in a dramatic reduction of tumor weight as compared with vehicle-treated control mice after just 72 hours (*P* = 0.04, Figure 2D). Extended brequinar treatment in TH-*MYCN* mice (120 days) led to a strikingly prolonged survival (*P* < 0.0001, Figure 2E). Similar to the SK-N-BE(2)C xenografts, tumors eventually relapsed after discontinuation of brequinar treatment (Supplemental Figure 2G).

### MYCN and DHODH dependencies correlate in neuroblastoma.

**Figure 3:**
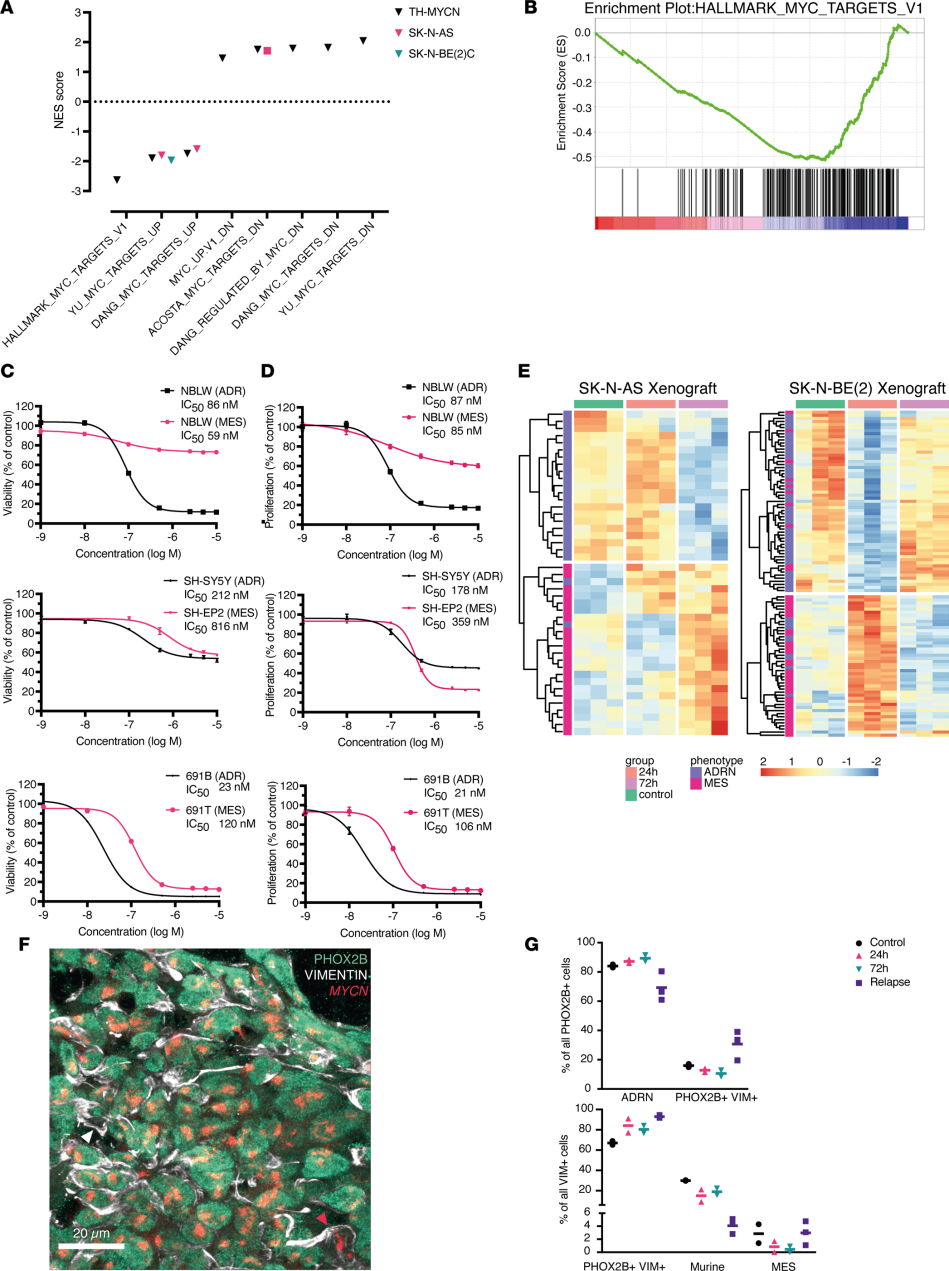
Brequinar inhibits MYC target gene expression and induces ADRN/MES transitions in vivo. (**A**) Normalized enrichment scores (NESs) of significantly deregulated MYC target genes from gene set enrichment analysis (GSEA) of RNA-Seq data in brequinar-treated xenografts and transgenic mice. Triangles represent tumors 72 hours after 1 dose of brequinar; squares represent tumors 24 hours after 1 dose of brequinar. Gene set names from MSigDB (Hallmarks and CGP) are provided on the *x* axis. Negative NESs indicate significant downregulation; positive NESs indicate significant upregulation. MYC family target genes are downregulated after brequinar treatment, whereas genes downregulated by MYC increase after brequinar treatment. (**B**) GSEA enrichment plot demonstrating MYC target gene downregulation (MSigDB Hallmarks MYC target v1 gene set) in brequinar-treated TH-*MYCN* tumors. NES = –2.63, NOM *P* = 0.000, FDR *q* = 0.000. NOM, nominal. (**C** and **D**) MTT viability assay (**C**) and CyQuant proliferation assay (**D**) in 3 isogenic ADRN (black) and MES (pink) neuroblastoma cell line pairs. (**E**) Heatmaps showing *z* scores of significantly deregulated ADRN/MES genes in brequinar-treated xenografts: SK-N-AS at 72 hours; SK-N-BE(2) at 24 hours. (**F**) Example of increased VIM expression in relapsed SK-N-BE(2)C xenograft tumors following 18 days of brequinar treatment. Image represents maximum intensity projection of a *Z*-stack. Green color indicates PHOX2B staining (ADRN marker), white color indicates vimentin (VIM) staining (MES marker), and red color indicates *MYCN* FISH (human neuroblastoma cell marker). Scale bar indicates 20 μm. Red arrow indicates an MES cell (PHOX2B^–^*MYCN^+^*VIM^+^); white arrow indicates a murine stromal cell (PHOX2B^–^*MYCN*^–^VIM^+^). (**G**) Quantification of ADRN/MES transitions in brequinar-treated SK-N-BE(2)C xenografts at 24/72 hours and at relapse. One symbol represents 1 mouse. ADRN, PHOX2B^+^ VIM^–^*MYCN*^–^; murine, PHOX2B^–^*MYCN*^–^VIM^+^; MES, PHOX2B^–^VIM^+^*MYCN*^+^. Numbers of quantified PHOX2B^+^VIM^+^ cells per sample are provided in the Methods section.

Brequinar was highly effective in *MYCN-*driven neuroblastoma models in vivo. To study the transcriptomic effects of DHODH inhibition, we performed RNA-Seq on brequinar-treated xenograft tumors and TH-*MYCN* tumors. GSEA showed brequinar-induced downregulation of MYC target genes (Figure 3, A and B).

We next examined whether *DHODH* dependency correlates with *MYCN* dependency in neuroblastoma. We observed a significant correlation in CERES scores between these genes (Supplemental Figure 3A), where *MYCN-*dependent cell lines tended to be more dependent on *DHODH*. At 24 hours, brequinar treatment resulted in the downregulation of *DHODH* as well as *MYCN* [SK-N-BE(2)C] or *MYC* [SK-N-AS]. (Supplemental Figure 3B). In comparison with SK-N-BE(2)C, SK-N-AS cells recovered their *MYC* and *DHODH* expression, suggesting this may be a potential mechanism underlying their relative resistance to DHODH inhibition. A similar phenomenon was observed in brequinar-treated xenografts, where *MYCN* [SK-N-BE(2)C] or *MYC* [SK-N-AS] expression recovered 72 hours posttreatment (Supplemental Figure 3C). In the TH-*MYCN* mouse model, we observed that endogenous *Mycn* was reduced 72 hours after 1 dose of brequinar. However, expression of the human *MYCN* transgene was not affected after 72 hours (Supplemental Figure 3D). This may be comparable to the recovery effect of *MYCN* expression seen after 72 hours in SK-N-BE(2)C xenografts (Supplemental Figure 3C).

### DHODH inhibition preferentially targets the neuroblastoma ADRN cell state.

Neuroblastoma cell lines exist in at least 2 distinct malignant cellular states: MES and ADRN cells (16, 17). In light of this phenomenon, we analyzed gene expression data from CCLE neuroblastoma cell lines and generated ADRN and MES scores using the expression signature scoring as described by van Groningen et al. (16). A group of 5 cell lines (SK-N-SH, LS, SK-N-AS, CHP-212, and GI-ME-N) displayed MES transcriptional profiles with high MES scores (Supplemental Figure 3E) while displaying high *DHODH* CERES scores, indicating a lower likelihood of *DHODH* dependency. Conversely, the neuroblastoma cell lines that were most dependent on *DHODH* all had low MES scores (Supplemental Figure 3E). Furthermore, SK-N-AS and SH-EP cells were also the most resistant to treatment with brequinar in vitro (Supplemental Figure 2A), with the sensitivity of SH-EP cells comparable to the nonmalignant stromal MRC5 cell line (Supplemental Figure 2, A and B). Of note, SH-EP is known to display an MES phenotype (16, 17). Moreover, in SK-N-AS cells (high MES score, Supplemental Figure 3E), we observed a plateau effect where increasing doses of brequinar did not further decrease cell viability (Supplemental Figure 2A), suggesting that neuroblastoma cells with a predominantly MES phenotype are less dependent on DHODH and more resistant to its inhibition.

To compare the sensitivity to brequinar within the same cell line model, we treated isogenic cell line pairs that reflect ADRN or MES cell states: SH-EP2/SH-SY5Y, 691B/691T, and NBLW-ADRN/NBLW-MES (16). We demonstrated that the ADRN neuroblastoma cells were more sensitive to DHODH inhibition in comparison with their MES counterparts (Figure 3, C and D), further substantiating our observation that the MES cell state is more resistant to DHODH inhibition.

To explore this phenomenon in vivo, we treated mice bearing SK-N-BE(2)C (ADRN phenotype) and SK-N-AS (MES phenotype) xenografts with a single dose of brequinar and harvested tumors after 72 hours. Relapsed tumors (after discontinuation of brequinar treatment) were also sampled for analysis. We observed a modest transcriptomic shift toward a more MES-like signature after brequinar treatment (Figure 3E and Supplemental Figure 3F). In SK-N-AS xenografts, the shift from ADRN toward MES signatures was evident at 72 hours (Supplemental Figure 3F). In SK-N-BE(2)C xenografts, the ADRN scores transiently decreased at 24 hours but were comparable to controls 72 hours after 1 dose of brequinar (Supplemental Figure 3F). Among the ADRN/MES signature genes that were significantly deregulated at 24 [SK-N-BE(2)C] or 72 [SK-N-AS] hours, upregulated genes were mainly MES signature genes, whereas downregulated genes were mainly ADRN signature genes in both cell lines (Figure 3E). Taken together, we observed an increased MES and/or decreased ADRN signature after DHODH inhibition in vivo. This may be explained by brequinar preferentially inhibiting ADRN tumor cells, shifting the balance between these 2 populations in the tumor tissue.

To validate these findings, we performed a combination of fluorescence in situ hybridization (FISH) and immunofluorescence staining on brequinar-treated SK-N-BE(2)C xenograft tumors at 24 and 72 hours after 1 dose (Figure 3, F and G, and Supplemental Figure 7). We also stained relapsed xenograft tumors (after 18 days of brequinar treatment). We used a *MYCN* FISH probe as a marker for human malignant SK-N-BE(2)C cells, PHOX2B as an ADRN marker, and VIM as a marker for MES cells (Figure 3, F and G) (16). Using this method, we identified an increased number of PHOX2B^+^VIM^+^ double-positive neuroblastoma cells after brequinar treatment and at relapse. At the time of relapse, the number of double-positive cells was clearly increased. This fits well with previous descriptions of the MES neuroblastoma signature being enriched in relapsed samples (16–18). However, relapses did not display a “full” shift toward the MES state as the number of MES cells (PHOX2B^–^VIM^+^*MYCN^+^*) was comparable to controls (Figure 3G and Supplemental Figure 7).

We next evaluated the super-enhancer landscape of brequinar-treated TH-*MYCN* tumors using ChIP-Seq to assess H3K27Ac signal at 72 hours after 1 dose of brequinar. Overall, brequinar-treated and control samples were highly similar in terms of their enhancer profiles (Supplemental Figure 4, A–C), and super-enhancers typical of the ADRN neuroblastoma phenotype (16) were primarily observed in both groups (Supplemental Figure 4C). H&E stainings of brequinar-treated TH-*MYCN* tumors indicated that the amount of stroma relative to malignant cells was increased, in particular after 48 hours (Supplemental Figure 4D). Thus, a potential super-enhancer–driven shift from the ADRN toward the MES state after brequinar treatment was not evident in TH-*MYCN* tumors.

Ferroptosis, programmed cell death characterized by mitochondrial lipid peroxidation, is a key tumor suppressor mechanism (32). Glutathione peroxidase 4 (GPX4) is a major cellular defense system against ferroptosis. It has recently been demonstrated that GPX4 activity is mediated by DHODH and that brequinar suppresses tumor growth of GPX4^lo^ cells by inducing ferroptosis (27). We hypothesized that brequinar may induce antitumor ferroptosis in neuroblastoma cells in vivo. To address this, we performed GSEA on RNA-Seq data from short-term–treated xenografts [SK-N-BE(2)C, SK-N-AS] and transgenic TH-*MYCN* mice. Ferroptosis-related genes were significantly upregulated in TH-*MYCN* (NES 1.41, NOM *P* value 0.032, *q* value 0.089) and SK-N-AS xenografts (NES 1.46, NOM *P* value 0.018, FDR *q* value 0.236) (Supplemental Table 5). Although *GPX4* expression levels were similar between SK-N-AS, SK-N-BE(2)C and TH-*MYCN* tumors (Supplemental Figure 3G), the significant upregulation of ferroptosis-related genes may explain part of the brequinar-induced antitumor activity in vivo.

### A combination of DHODH inhibition and temozolomide demonstrates curative potential in transgenic neuroblastoma mice.

Given that single-agent DHODH inhibition was not curative in the xenograft and transgenic in vivo models of neuroblastoma, we next sought to combine brequinar with the standard-of-care chemotherapy temozolomide, a DNA alkylating agent, which is the backbone of treatment in relapsed and refractory neuroblastoma (33).

**Figure 4:**
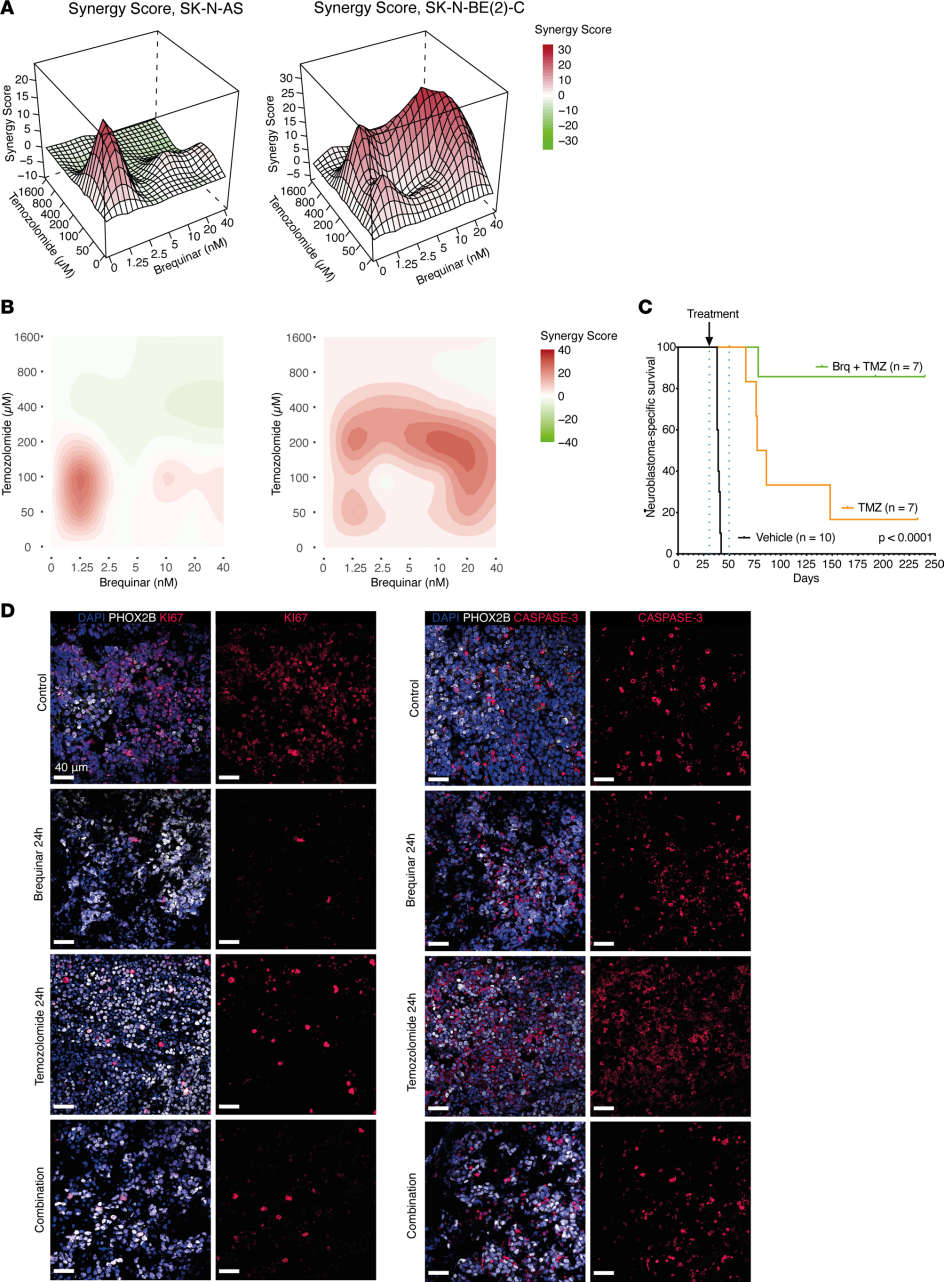
Combinations of brequinar and temozolomide are synergistic in vitro and have curative potential in vivo. (**A** and **B**) Synergy scores of temozolomide and brequinar combinations in SK-N-AS (**A**) and SK-N-BE(2) (**B**) cells treated with both drugs. Scores are based on 3 independent experiments. Red color indicates synergy. ZIP scores are shown as 3D (**A**) and 2D (**B**) representations. ZIP scores indicate the average percentage excess response due to drug interactions. (**C**) Overall survival of homozygous TH-*MYCN* mice treated with either temozolomide alone (*n* = 7) or a combination of brequinar and temozolomide (*n* = 7). For comparison, control mice (shown in black) are the same as in Figure 2E. Groups are compared using log-rank test. (**D**) Immunostainings at original magnification 40× of PHOX2B (white), the apoptosis marker active caspase-3 (red), and the proliferation marker Ki67 (red) in TH-*MYCN* tumors treated with 1 dose of brequinar and/or temozolomide, sampled 24 hours after the last dose. Nuclei were counterstained with DAPI (blue). Scale bar indicates 40 μm.

Brequinar and temozolomide demonstrated a synergistic inhibitory effect in vitro (Figure 4, A and B; and Supplemental Figure 5, A–D). To explore this effect in vivo, we treated tumor-bearing homozygous TH-*MYCN* mice with 1 dose of brequinar and/or temozolomide and performed tumor immunohistochemistry on tumors labeling proliferating cells (Ki67) and apoptosis (active caspase-3). At 24 hours, after 1 dose of either brequinar or temozolomide, we observed a marked decrease in the proportion of Ki67^+^ cells and an increase in active caspase-3^+^ cells (Figure 4D). At 24 hours after 2 consecutive days of treatment, first with brequinar, then temozolomide, we observed a strong increase in cells expressing the MES/stromal marker VIM (Supplemental Figure 5E).

To investigate the therapeutic efficacy of this promising combination, we treated homozygous TH-*MYCN* mice with 3 doses of brequinar followed by 2 cycles of temozolomide or with 2 cycles of temozolomide alone (Supplemental Figure 6A). The TH-*MYCN* mice previously treated with vehicle control were used as a comparator group (Figure 2E and Figure 4C), and these untreated mice generally succumb within approximately 6 weeks postbirth. When comparing temozolomide- and combination-treated with vehicle-treated mice, survival was significantly prolonged in both groups (*P* < 0.0001) (Figure 4C). However, following discontinuation of treatment, all but 1 of the temozolomide-treated mice had a relapse. In the combination group, we observed a dramatic prolongation of survival where 5 out of 7 mice did not experience neuroblastoma relapse even 190 days after treatment cessation (Figure 4D). Two mice in the combination therapy group ultimately died from thoracic tumors, of which 1 was a PHOX2B^+^ thoracic neuroblastoma (suggesting a metastatic or primary thoracic neuroblastoma) and the other a PHOX2B^–^MYCN^–^CD45^+^CD3^+^ lymphoma (Supplemental Figure 6, B and C). In conclusion, the combination of brequinar and temozolomide has curative potential in vivo.

## Discussion

In patients with high-risk neuroblastoma, there is currently a need for improved therapy, as survival rates remain less than 50% (34). The aim of this study was to identify metabolic vulnerabilities in neuroblastoma and to evaluate the role of DHODH as a potential therapeutic target in this disease. Brequinar was first introduced as an anticancer agent in the 1980s (35). Several phase II clinical trials were performed in the following decade, evaluating the therapeutic potential of DHODH inhibition in a wide range of adult solid tumor malignancies (36–39). Although brequinar was well tolerated, its clinical efficacy was disappointing. Later, the DHODH inhibitor leflunomide was approved as an antiinflammatory agent in the treatment of rheumatoid arthritis. Interestingly, DHODH inhibition with leflunomide has been shown to block neural crest development and decrease melanoma growth in vivo (20). In addition, leflunomide has been explored as a potential therapeutic agent in the treatment of multiple myeloma (40). This is of potential interest for neuroblastoma research as both neuroblastoma and melanoma are neural crest–derived cancers (1, 41). Using a large high-throughput chemical screen, we previously identified that DHODH inhibitors were capable of inducing differentiation in AML models in vitro and in vivo (26).

By analyzing publicly available, large neuroblastoma patient cohorts, we presented *DHODH* expression as an independent prognostic marker in neuroblastoma after adjusting for known adverse factors, such as disease stage, age, and *MYCN* amplification. Furthermore, we observed a subset of high-risk, non–*MYCN*-amplified tumors with very high *DHODH* expression levels and particularly dismal prognoses. This indicated a potential biological role of DHODH in neuroblastoma and prompted us to perform further analyses to evaluate DHODH as a therapeutic target.

In this study, neuroblastoma cells were highly sensitive to DHODH inhibition, with IC_50_ concentrations in the low nanomolar range. Additionally, we observed that the most sensitive cell lines, such as SK-N-BE(2)C, were the ones with highly ADRN phenotypes (16, 17) and that *MYCN* dependency correlated with *DHODH* dependency. In contrast, cell lines with MES phenotype, such as SK-N-AS and SH-EP, were more resistant to DHODH inhibition.

When comparing ADRN/MES cell line pairs, we also observed that MES cell lines were more sensitive to DHODH inhibition than their ADRN counterparts (Figure 3, C and D). However, the difference in brequinar response was less pronounced between the SH-SY5Y and SH-EP2 cell lines. There was also an observed discrepancy between the MTT viability and CyQuant proliferation assays in terms of sensitivity to brequinar: in the former, ADRN phenotype cells were more sensitive, whereas in the latter, MES phenotype cells were more sensitive to DHODH inhibition. 691-MES/691-ADRN and NBLW cell lines are all *MYCN* amplified (16, 42). SH-SY5Y harbors a chromosomal gain at 2p, which includes the *MYCN* locus, whereas SH-EP2 does not (43). As CERES/DepMap results indicated that *DHODH* dependency correlated with *MYCN* dependency (Supplemental Figure 3A), it is possible that the difference in brequinar response between SH-SY5Y and SH-EP2 cell lines is influenced by the differing *MYCN* activity as well as the ADRN/MES phenotype difference.

When we compared the *MYCN*-amplified cell line SK-N-BE(2)C with the non–*MYCN-*amplified SK-N-AS cell line, we observed a greater reduction of pyrimidines UTP and CTP upon brequinar treatment in the SK-N-BE(2)C cells. In addition, using shRNA *DHODH* knockdown, we observed an increase in cleaved PARP and activated caspase-3 compared with shGFP control in SK-N-BE(2)C but not SK-N-AS cells. Similar observations were made after treatment with brequinar, suggesting increased apoptosis in SK-N-BE(2)C cells as a result of DHODH knockdown or inhibition due to reduction of UTP and CTP pyrimidines. This might explain our observation that SK-N-AS cells were more resistant to brequinar in vitro. Furthermore, GSEAs of RNA-Seq data from mouse xenografts and transgenic TH-*MYCN* tumors demonstrated that brequinar treatment caused a reduction of *MYCN/MYC* expression and MYC targets in vivo. Taken together, these results may indicate that *MYCN*-driven neuroblastoma cells are less capable of pyrimidine salvage and more dependent on de novo pyrimidine biosynthesis via DHODH. However, even though DHODH inhibition was effective in the ADRN, *MYCN*-driven SK-N-BE(2)C cells in vivo, monotherapy was not curative, and DHODH inhibition did not induce terminal differentiation.

The TH-*MYCN* model is a model of spontaneous and aggressive neuroblastoma that is also driven by *MYCN* overexpression (31). In this model, DHODH inhibition was also highly effective in preventing tumor growth. However, similar to SK-N-BE(2)C xenografts, the effect was incomplete, and in both model systems, tumors relapsed 1–2 weeks following the discontinuation of treatment.

Brequinar treatment triggered a modest transcriptomic shift from an ADRN signature toward an MES signature in vivo. At the time of relapse, we observed an increased number of VIM^+^ tumor cells, indicating ongoing transitions toward the MES state. This fits well with previous observations by others (18). However, neither short-term brequinar treatment (1 dose) nor 18-day therapy was sufficient to push the cells into a terminal or stable MES state, though an increased number of transitioning cells was observed in the relapses in particular. This may be explained by brequinar preferentially killing the “pure” ADRN cells, resulting in a higher residual number of MES neuroblastoma cells.

The combination of the alkylating agent temozolomide and the DHODH inhibitor brequinar demonstrated synergistic effects in vitro and a striking effect on the survival of homozygous transgenic TH-*MYCN* mice. Temozolomide is approved and in clinical use in the relapse setting, hence providing a rationale for clinical use with DHODH inhibitors. Pyrimidine depletion has previously been shown to induce DNA damage and double-strand breaks (44). Thus, one possible explanation for the observed synergy may be the combined effect of alkylation and pyrimidine depletion on DNA damage and breakage.

In conclusion, our data demonstrate the therapeutic impact of DHODH as a critical mediator of neuroblastoma cell growth. Specific DHODH inhibition combined with the alkylating agent temozolomide is highly effective in neuroblastoma preclinical models. Targeting DHODH as a cancer-specific metabolic dependency may benefit high-risk neuroblastoma patients and is a strong candidate for further testing in clinical studies.

## Methods

Additional details are in Supplemental Methods.

### Cell cultures and reagents.

For WST-1 cell viability and quantitative PCR (qPCR) analyses, cell lines were grown in DMEM/F12 [Gibco, Thermo Fisher Scientific; SH-SY5Y] or RPMI 1640 [Gibco, Thermo Fisher Scientific; SK-N-AS, SK-N-BE(2)C, SK-N-FI, SK-N-DZ, SK-N-SH, IMR-32, Kelly, SH-EP, MRC5] supplemented with 10% fetal bovine serum, 2 mM l-glutamine, 100 μg/mL streptomycin, and 100 IU/mL penicillin G (all from Life Technologies, Thermo Fisher Scientific). Cell lines were purchased from ATCC (ATCC-LGC Standards) and grown at 37°C in a humidified 5% CO_2_ atmosphere. Cell line identities were verified by short tandem repeat genetic profiling (AmpFISTR Identifiler PCR Amplification Kit; Applied Biosystems, Thermo Fisher Scientific), routinely tested for mycoplasma (Mycoplasmacheck, Eurofins Genomics), and used in passages below 25.

For MTT viability and CyQuant proliferation analyses, SH-SY5Y and SH-EP2 cell lines were cultured as previously described (45). The isogenic cell line pair 691B and 691T was generated and cultured in neural stem cell medium as previously described (46). The NBLW-ADR and NBLW-MES cell lines were derived from the parental NBL-W cell line (42) through their differential adhesion to culture plates. All NBL-W cell lines were cultured in RPMI 1640 medium supplemented with 10% fetal bovine serum, 1× nonessential amino acids, 20 mM l-glutamine, 10 U/mL penicillin, and 10 μg/mL streptomycin (all from Life Technologies, Thermo Fisher Scientific). Cell line identities of isogenic ADRN/MES cell line pairs were verified by short tandem repeat analysis (Multiplexion). Cell lines were routinely checked for the presence of mycoplasma using the MycoAlert detection kit (Lonza).

### DHODH knockdown and inhibition.

The experiments were performed as described previously (47). For shRNA-mediated *DHODH* knockdown, lentiviral shRNA constructs shDHODH-32 (TRCN0000025832) and shDHODH-68 (TRCN0000025868) were obtained from MilliporeSigma. Lentiviruses for shRNA expression were produced in 293FT cells (Thermo Fisher Scientific) using the packaging plasmids pLP1, pLP2, and pLP/VSVG (Thermo Fisher Scientific, K497500), and lentiviral infections of cells were conducted according to standard procedures. For DHODH inhibition in transfected cell lines, brequinar sodium (Tocris, Bio-Techne, 96201-88-6) and GSK983 (MilliporeSigma SML1824) were dissolved in DMSO (Thermo Fisher Scientific BP231-100), and stock solutions were aliquoted and stored at –80°C until use. Cells were treated with DMSO, brequinar, or GSK983 at the indicated concentrations and times and then collected for immunoblotting analysis.

For immunoblotting, proteins (approximately 25 μg) were separated on SDS-PAGE, transferred to nitrocellulose membranes (926-31092, LI-COR Biosciences), and probed with the following primary antibodies: mouse anti-DHODH (Santa Cruz Biotechnology, clone E8, catalog number sc-166348, 1:400), rabbit anti-cleaved PARP (Cell Signaling Technologies, clone D64E10 XP, catalog number 5625, 1:1,000), rabbit anti–cleaved caspase-3 (Cell Signaling Technologies, polyclonal, catalog number 9661, 1:1,000), and mouse anti–α-tubulin (MilliporeSigma, clone B-5-1-2, catalog number T5168, 1:5,000). Horseradish peroxidase–conjugated goat anti-mouse (Santa Cruz Biotechnology, catalog number sc-2005) and anti-rabbit IgG (Santa Cruz Biotechnology, catalog number sc-2004) were used as secondary antibodies. Proteins were visualized using a Clarity Western ECL Kit (Bio-Rad) and Amersham ImageQuant 800 imager (Cytiva).

### Drugs.

For in vitro viability assays and qPCR studies, brequinar (MilliporeSigma, catalog SML0113) and temozolomide (MilliporeSigma, catalog T2577) were dissolved in DMSO. For in vivo studies, brequinar was synthesized as described previously (26) and dissolved in a vehicle consisting of 70% PBS and 30% polyethylene glycol–400 (PEG-400), pH 7.2, while temozolomide (MilliporeSigma, catalog SML0113) was dissolved in DMSO and further diluted in sterile physiological saline.

### Cell viability and proliferation assays.

For WST-1 cell viability analyses, Opti-MEM (Gibco, Thermo Fisher Scientific) was used and supplemented with 1% fetal bovine serum and penicillin, streptomycin, and l-glutamine in the same concentrations as for cell culturing. Cells were seeded in 96-well plates [SK-N-BE(2)C: 5,000 cells per well; all other cell lines: 10,000 cells per well]. The following day, cells were treated with brequinar and/or temozolomide. After 72 hours, cell viability was evaluated using a colorimetric formazan-based cell viability assay (WST-1; Roche, MilliporeSigma). Absorbance was measured at 450 and 650 nm using a VersaMax reader (Molecular Devices). Cell viability is presented as percentage of untreated control cells. All concentrations were at least tested in triplicates and experiments repeated at least 3 times. IC_50_ values (inhibitory concentration 50%) were calculated from log concentration-effect curves in Prism (GraphPad Software) using nonlinear regression analysis. For temozolomide/brequinar combination experiments in vitro, viability concentrations (percentage of control) with corresponding drug concentrations were analyzed using the *synergyfinder* R package (v2.4.16) (48).

For CyQuant proliferation and MTT viability assays, SH-SY5Y/SH-EP2 (DMEM; 10,000/5,000 cells per well), 691B/691T (culturing medium as described above; 15,000 cells per well), and NBLW-ADRN/NBLW-MES (RPMI 1640; 15,000/7,500 cells per well) cell line pairs were seeded in 96-well plates and treated with brequinar in the indicated concentrations. For each drug concentration, sextuplet replicates were used. After 72 hours of treatment, cell DNA content was measured using the CyQuant assay (C35012, Life Technologies, Thermo Fisher Scientific) according to the manufacturer’s instructions with the exception that CyQuant reagents were added at half of the indicated volumes. Metabolic activity was measured by adding 10 μL of MTT (10 mg/mL, MilliporeSigma). After 4 hours of incubation, 100 μL of 10% SDS/0.01 M HCl was added to stop the reaction. Optical densities were quantified using a Synergy HT microplate reader (BioTek, Agilent).

### Incubation of cells with drug for metabolite measurement.

SK-N-BE(2)C and SK-N-AS cells were incubated with 1 μM of brequinar for different times. A total of 500,000 cells were harvested for each sample and cell line respectively at the indicated times. After being washed with PBS, cells were processed for nucleotide extraction. Nucleotides were extracted using 0.4 M perchloric acid, and the extracts were neutralized with 10 M KOH and stored at –20°C until analyzed as described previously (49).

### Measurement of intracellular nucleoside triphosphate by HPLC.

The neutralized extracts were applied to an anion exchange Partisil 10 SAX column (MilliporeSigma) and eluted at a flow rate of 1.5 mL/min with a 50-minute concave gradient (curve 9, Alliance e2695 Separations Module, Waters) from 60% 0.005 M NH_4_H_2_PO_4_ (pH 2.8) and 40% 0.75 M NH_4_H_2_PO_4_ (pH 3.8) to 100% 0.75 M NH_4_H_2_PO_4_ (pH 3.8). The column eluate was monitored by UV absorption at 262 nm, and the nucleoside triphosphates were quantitated by electronic integration with reference to external standards. The intracellular concentration of nucleotides contained in the extract was calculated from a given number of cells of a determined mean volume. The calculation assumed that the nucleotides were uniformly distributed in a total cell volume.

### Animal studies.

Female NMRI *nu/nu* mice (purchased from Scanbur) of 4 to 6 weeks of age were injected with 5 million SK-N-AS or SK-N-BE(2)C cells subcutaneously in the flank and monitored daily for tumor growth. Tumors were measured by caliper and tumor volume was calculated using the formula (width)^2^ × length × 0.44. For each tumor, tumor volume index (TVI) was defined as tumor volume relative to inclusion volume. TVI was calculated each day until the first control was sacrificed. When tumors were detectable and tumor volume exceeded 200 mm^3^, mice were block randomized into 2 groups (treatment or control). Mice in the treatment group received 50 mg/kg brequinar every third day by intraperitoneal injection for a maximum of 7 doses. The animals were monitored daily for signs of toxicity, including weight loss. Mice were sacrificed when the tumor reached a total volume of 2,000 mm^3^ or the tumor exceeded length or width of 20 mm. After mouse sacrifice, tumors were dissected in smaller parts, then flash frozen, fixed in 4% paraformaldehyde or stored in RNAlater (Invitrogen, Thermo Fisher Scientific).

Transgenic TH-*MYCN* mice were obtained from the Mouse Model of Human Cancer Consortium repository and kept as an inbred colony on the 129X1/SvJ background (The Jackson Laboratory). Genotypes were determined from ear tissue biopsies using qPCR with specific probes designed for wild-type and the *MYCN* transgene (Transnetyx). Only *MYCN*-homozygous mice (of either sex) were used for in vivo studies. In the first treatment study (Figure 2E), homozygous mice at 32 days of age were block randomized into 2 groups, which received intraperitoneal injections of either vehicle (70% PBS, 30% PEG-400) (*n* = 10) or brequinar (50 mg/kg) every third day (*n* = 11) for 120 consecutive days. In the second experiment (Figure 4C), at 32 days of age, homozygous mice were block randomized into 2 groups: the combination group (*n* = 7) or the temozolomide group (*n* = 7). Mice in the combination group were treated with brequinar every third day (50 mg/kg) for a total of 3 doses (treatment day 0, day 3, day 6) followed by 20 mg/kg temozolomide intraperitoneally for a total of 10 doses (treatment days 7–11 and 14–18). Mice in the temozolomide group were treated with temozolomide intraperitoneally (20 mg/kg) at treatment days 0–4 and 7–11 for a total of 10 doses. For time point sampling of homozygous TH-*MYCN* tumors subjected to single-agent treatment, mice with palpable abdominal tumors received 1 intraperitoneal dose of temozolomide (20 mg/kg), brequinar (50 mg/kg), or vehicle (70% PBS, 30% PEG-400). Mice were then sacrificed and tumors were sampled after 6, 24, 48, or 72 hours. For sampling of homozygous TH-*MYCN* tumors subjected to combination treatment, mice with palpable abdominal tumors received 1 intraperitoneal dose of brequinar (50 mg/kg) on day 0 and 1 intraperitoneal dose of temozolomide (20 mg/kg) on day 1, and mice were sacrificed on day 2. All animals were maintained at a maximum of 6 animals per cage and given sterile water and food ad libitum.

### Sectioning, FISH, and immunofluorescence staining of murine neuroblastoma tumors.

Tumors from TH-*MYCN* mice and SK-N-BE(2)C NMRI *nu/nu* xenografts were dissected and cut into smaller pieces (3 × 3 × 3 mm). The tumor pieces were washed in ice-cold PBS and fixed in 4% paraformaldehyde in PBS (pH 7.4) at 4°C for 1 hour. Fixed tumor pieces were washed in PBS and cryoprotected in 30% sucrose in PBS at 4°C for 3 hours with gentle agitation. Subsequently, tumor samples were embedded in embedded in OCT (Sakura Finetek) and frozen at –80°C. Each tumor sample was sectioned (12 μm) using Leica CM3050S (chamber temperature of –20°C, object temperature of –15°C), collected on SuperFrost Plus Adhesion microscope slides (Thermo Fisher Scientific), and stored at –20°C.

FISH was applied to SK-N-BE(2)C xenograft tumor sections for detection of *MYCN* amplification. Cryosection slides were brought to room temperature to dry for at least 1 hour, rehydrated in PBS for 15 minutes, and incubated in 0.5% Triton X-100 (Thermo Fisher Scientific) in PBS for 20 minutes at room temperature. Antigen retrieval was then performed by incubating sections in 10 mM sodium citrate buffer (pH 6), first for 10 minutes at room temperature, then at 80°C for 20 minutes. After cooling to room temperature, sections were washed in 2× saline sodium citrate (SSC) buffer, 3 times, for 5 minutes each. Sections were then incubated in 50% formamide in 2× SSC for 4 hours at room temperature. FISH probes for the *MYCN* gene labeled with 5-TAMRA (Empire Genomics) were diluted 1:5 in hybridization buffer (Empire Genomics), applied to sections, covered with coverslips, and sealed with rubber cement for hybridization. Slides were then incubated in a dark, humid chamber at 37°C for 1 hour. Next, slides were incubated at 80°C for 5 minutes, followed by hybridization at 37°C for 48–72 hours. After hybridization, sections were washed in 2× SSC, 3 times, for 5 minutes each at 37°C; then in 0.1× SSC, 3 times, for 5 minutes each at 60°C. The sections were then immunostained for PHOX2B and VIM, without the additional epitope retrieval step.

For immunostaining without FISH, the slides with tumor sections were removed from –20°C and brought to room temperature to dry for 30 minutes. Heat-induced target epitope retrieval was performed by heating 1× Target Retrieval Solution (Dako) to 99°C, submerging slides into near-boiling Target Retrieval Solution, then allowing for it to cool down to room temperature. Slides were washed in PBS containing 0.1% Tween 20 (PBS-T) 3 times, 10 minutes each. Primary antibodies diluted in PBS-T were added to the sections and incubated overnight at 4°C in a dark, humid chamber. Sections were then washed in PBS-T 3 times, for 10 minutes each, followed by incubation with secondary antibodies with DAPI diluted in PBS-T for 90 minutes at room temperature, in a dark, humid chamber. Finally, sections were washed in PBS-T 3 times, for 10 minutes each, and then mounted using Mowiol mounting medium (Merck). The primary antibodies used were PHOX2B (R&D Systems, Bio-Techne; polyclonal, catalog number AF4940; 1:40), Ki67 (Invitrogen, Thermo Fisher Scientific; clone SP6, catalog number MA5-14520; 1:250), active caspase-3 (R&D Systems, Bio-Techne; polyclonal, catalog number AF835; 1:50), and VIM (Cell Signaling Technologies, clone D21H3, catalog number 5741; 1:100). For primary antibody detection, secondary antibodies were used, raised in donkey against goat or rabbit conjugated with Alexa Fluor 488 or 555 fluorophores (Invitrogen, Thermo Fisher Scientific; both polyclonal, catalog numbers A32814, A32794) in 1:1,000 dilutions. Immunostaining images were acquired using LSM700 confocal microscope (Zeiss) with 40× (water) and 63× (oil) objectives. Images were acquired in.lsm format, and Imaris software was used for further analysis.

For quantification of PHOX2B/VIM/*MYCN* FISH stainings, 63× *Z*-stack images were analyzed. The total numbers of quantified cells were as follows: control 1: 302 PHOX2B^+^ cells total, 69 VIM^+^ cells total. Control 2: 589 PHOX2B^+^ cells total, 145 VIM^+^ cells total. 24h 1: 612 PHOX2B^+^ cells total, 78 VIM^+^ cells total. 24h 2: 652 PHOX2B^+^ cells total, 118 VIM^+^ cells total. 72h 1: 830 PHOX2B^+^ cells total, 126 VIM^+^ cells total. 72h 2: 1,006 PHOX2B^+^ cells total, 112 VIM^+^ cells total. Relapse 1: 1,094 PHOX2B^+^ cells total, 462 VIM^+^ cells total. Relapse 2: 1,043 PHOX2B^+^ cells total, 370 VIM^+^ cells total. Relapse 3: 920 PHOX2B^+^ cells total, 196 VIM^+^ cells total.

### RNA-Seq.

Mouse tumor tissue used for RNA sequencing was stored in RNAlater solution at –80°C. For brequinar-treated TH-*MYCN* tumors, tumor tissue RNA/DNA extraction, library preparation, and RNA/ChIP-Seq were performed by Active Motif. Briefly, total RNA was isolated using an RNeasy Mini Kit (QIAGEN). RNA quality was assessed using BioAnalyzer 2100 and/or TapeStation RNA Screen Tape (Agilent) and Qubit fluorometric assay (Thermo Fisher Scientific), with RNA integrity number values ranging from 6.8 to 9.4. Then, 42 bp paired-end libraries were prepared using the TruSeq stranded protocol and sequenced on the NextSeq 500 platform (Illumina) for a depth of 44.1–56.1 million read pairs.

Total RNA was extracted from SK-N-AS and SK-N-BE(2)C xenograft tissue using the RNeasy Mini Kit, and quality control was performed with TapeStation according to the manufacturer’s instructions. Libraries were prepared using the TruSeq Stranded mRNA protocol. Library quality control was performed using Qubit (Thermo Fisher Scientific) and Agilent TapeStation platform. Indexed libraries were sequenced on the NextSeq 500 platform (Illumina), generating 75 bp single-end reads. Base calling and demultiplexing were performed using CASAVA software with default settings.

### Bioinformatic analyses: ChIP-Seq.

ChIP-Seq analysis of TH-*MYCN* tumor samples was performed by Active Motif. For enhancer analyses, 75 bp reads were mapped to the mouse genome using the BWA algorithm with default settings. Duplicate reads were removed and only uniquely mapped reads with mapping quality ≥ 25 were used for further analyses. Using in-house software, alignments at 3′ ends were extended in silico to a length of 200 bp, which was the average genomic fragment length of the size-selected libraries. Extended alignments were assigned to 32 nt bins across the genome, and the number of fragments per bin was determined. H3K27Ac peaks were determined using the MACS algorithm (v2.1.0) (50) with *P* value cutoff 1 × 10^–7^. Peaks in the ENCODE exclusion list of known false ChiP-Seq peaks were filtered out. Tag numbers of all samples within a comparison group (i.e., controls or brequinar-treated samples) were reduced by random sampling to the number of tags present in the smallest sample. To identify super-enhancers, a proprietary algorithm was used. MACS peaks were merged if their inner distance was ≤ 12,500 bp. The 5% merged peak regions with the strongest signals were identified as super-enhancers. For gene annotation, genes within 25 kb of the super-enhancer regions were included.

### Bioinformatic analyses: RNA-Seq.

Raw sequencing.fastq files from xenografts and TH-*MYCN* tumors were aligned to a combined mm10/hg19 genome (10x Genomics, release 3.0.0). Raw reads were aligned using the STAR 2-pass approach (51). For the TH-*MYCN* data, any reads mapping to human genes other than *MYCN* were discarded. For the xenograft data, any reads mapping to murine genes were discarded. Aligned reads were quantified using htseq-count (52). Differential gene expression analyses were performed using DESeq2 (53). GSEAs were performed using GSEA (54) with normalized gene expression values in the.gct format and MSigDB gene sets v7.4 (55) as input. To quantify ADRN and MES scores, gene signatures and the scoring approach outlined by van Groningen et al. (16) was used.

### CCLE, DepMap, and TARGET data analysis.

Clean liquid chromatography-mass spectrometry metabolomics data of CCLE cell lines was acquired from the publication by Li et al. (56). Metabolomics analyses comparing neuroblastoma cell lines to other solid tumor cell lines were performed using the Statistical Analysis module of MetaboAnalyst 5.0 (57). In partial least-squares discriminant analysis, cell lines were grouped as neuroblastoma (*n* = 17) or other (*n* = 746). DepMap/CCLE copy number data (21Q2 public data set) was used to define *MYCN* amplification, where cell lines with log_2_(relative to ploidy + 1) > 3 were considered amplified. Cell line gene expression (log_2_[TPM+1], 21Q2 public data set) and CRISPR (DepMap 21Q2 public+score, CERES) data sets were acquired via the DepMap portal and annotated according to the primary disease in the corresponding cell line metadata table. Precomputed statistics for CRISPR data were collected in the DepMap portal. Gene expression values from various primary pediatric cancer samples (log_2_[RPM+1], TARGET data set) were accessed and downloaded through the cBioPortal website (http://www.cbioportal.org). To quantify ADRN and MES scores, gene signatures and the scoring approach outlined by van Groningen et al. (16) were used.

### Patient survival analysis.

Survival and gene expression data from 2 neuroblastoma patient cohorts were collected from the publicly available data sets SEQC-498 (NCBI Gene Expression Omnibus GSE49711) and TARGET-NBL (fragments per kilobase of transcript per million mapped reads upper quartile–normalized expression data downloaded from https://portal.gdc.cancer.gov). For the TARGET data set, corresponding survival data were acquired from a previous publication (58). Gene expression values were log_2_-transformed in order to achieve a distribution closer to the Gaussian distribution. The distribution of continuous variables was evaluated using histograms and normal QQ-plots. Overall survival was defined as days from diagnosis until death from any cause or end of follow-up. Event-free survival was defined as days from diagnosis until disease progression or relapse, death from any cause, or end of follow-up, whichever came first. The association between survival and covariates was evaluated using Cox proportional hazards models. The linearity assumption of continuous covariates was evaluated by fitting models with restricted cubic splines, with 3 knots, using the function *rcs* in the R package *rms*. The effect of age at diagnosis was found to be nonlinear, and age was thus adjusted for by using restricted cubic splines with 3 knots, in order to adjust for the whole age effect. All survival analyses were performed using R version 3.5.0. The “highest risk” subset of non–*MYCN*-amplified, high-risk neuroblastoma cases (Figure 1, F and G) was identified by using the KaplanScan feature of the R2 database (https://r2.amc.nl) with the minimum group size set to 8.

### Data availability.

Raw and processed data from RNA- and ChIP-Seq experiments are available at the Gene Expression Omnibus (https://www.ncbi.nlm.nih.gov/geo/), SuperSeries accession number GSE209813.

### Statistics.

Quantitative experimental data are reported as mean with SEM unless otherwise specified. For comparison between 2 groups, 2-tailed Student’s *t* test was performed. Data from more than 2 groups were compared using 1-way ANOVA with multiple comparisons. For comparisons between 2 groups over several time points, Holm-Šidák multiple-comparison test was applied. Log-rank tests were used to compare survival between groups. Simple linear regression tests were used to study the relationship between various CERES gene scores. For dose-response curves and IC_50_ calculations, nonlinear regression analysis was performed, log(inhibitor) versus response, variable slope with 4 parameters. All statistical analyses were performed using GraphPad Prism version 9.4.1 or R version 4.2.0. Statistical significance was assumed for *P* values below 0.05, unless otherwise specified.

### Study approval.

The animal experiments were approved by the Stockholm (Sweden) ethics committee for animal research (reference numbers N231/14, N42/14, 5163-2019 and 13820-2019), appointed and under the control of the Swedish Board of Agriculture and the Swedish Court. The animal experiments presented herein were in accordance with national regulations (SFS no. 2018:1192 and SFS no. 2019:66).

## Author contributions

TKO, CD, DBS, and NB designed the study. TKO, CD, BE, AA, JM, JO, CT, IHM, JD, HFD, EMW, JK, RV, JIJ, PK, DBS, and NB performed data acquisition, analysis, and interpretation. TKO and CD drafted the manuscript. TKO, CD, DBS, and NB performed critical manuscript revision.

## Supplementary Material

Supplemental data

Supplemental table 1

Supplemental table 2

Supplemental table 3

Supplemental table 4

Supplemental table 5

## Version 1 08/09/2022

In-Press Preview

## Version 2 09/08/2022

Electronic publication

## Version 3 10/10/2022

Corrected publication date in PDF version
