# Supplementary material for: DHODH is an independent prognostic marker and potent therapeutic target in neuroblastoma
Source: JCI Insight. 2022 Sep 8;7(17):e153836. doi: 10.1172/jci.insight.153836 (PMC9798925; doi:10.1172/jci.insight.153836)
Supplement: Supplemental data [file jciinsight-7-153836-s005.pdf]

Supplementary Methods

CFSE analysis of proliferation rate

Cells were labeled per the manufacturer's protocol with CellTrace Far Red (Thermo Fisher Scientific). The cells were then assayed daily by flow cytometry to monitor the decay of their geometric mean fluorescence intensity. A viability dye (7-AAD) was included to exclude dead cells. The data were plotted using GraphPad Prism in order to calculate the fluorescence decay and doubling time.

Quantitative PCR

SK-N-BE (2) and SK-N-AS cells were treated with vehicle or brequinar (180 nM SK-N-AS, 7 nM SK-N-BE(2)) for 24 and 72 hours. Total RNA was prepared from cultured cells using the RNeasy MiniKit (Qiagen) including an on-column DNase treatment with the RNase-Free DNase Set (Qiagen). Isolated RNA was transcribed into cDNA with the High-Capacity RNA-to-cDNA Kit (Applied Biosystems). For amplification, the C100 Touch (Bio-Rad) thermocycler was used. mRNA level was assessed using the TaqMan Universal PCR Master Mix (Applied Biosystems) with the sequence-specific probes DHODH (Hs00361406_m1), MYCN (Hs00232074_m1), HPRT1 (Hs02800695_m1) and SDHA (Hs00188166_m1). Relative expression was calculated according to the delta-delta Ct Method normalized to housekeeping genes HPRT1 and SDHA. For *MYCN*, mRNA expression level was normalized to expression in the MRC5 fibroblast cell line. For relative *DHODH* expression, mRNA levels were normalized to untreated controls.

Flow cytometry

Control (shGFP) and DHODH knockdown (shDHODH) BE(2)-C and SK-N-AS cells were collected, washed with PBS, and fixed in 70% cold ethanol for at least 30 minutes. After fixation, cells were stained for 30 minutes in a PBS buffer containing 0.1% Triton X-100 (X100, Sigma-Aldrich), 200 µg/ml ribonuclease A (Qiagen 19101), and 20 µg/ml propidium

iodide (P3566, Thermo Fisher Scientific). Data were acquired using a BD LSRFortessa system (BD Biosciences) and analyzed with FlowJo v10 software.

<u>Immunohistochemistry</u>

Following resection, tumor tissue was fixed with formaldehyde (3.7-4.0% w/v, AppliChem) for 24h. Fixed tissues were processed in an automated tissue processor (LOGOS, Milestone) and embedded in paraffin. FFPE sections, 4 µm thick, were mounted on glass slides (Superfrost+, Thermo Scientific) and heated for 3 hours at 56 °C. Following de-paraffinization in Neo-Clear® (Sigma-Aldrich, Merck) for 2x15 min and rehydration in series of graded alcohols, HIER was performed in Target Retrieval Solution, Citrate pH 6.1 (Dako, Agilent) using a Decloaking Chamber (Biocare Medical) set to 5 min at 110 °C. Unspecific antibody binding sites were blocked with 5% goat serum (Sigma-Aldrich) in TBS containing 0.1% Tween 20 (TBST) (Sigma-Aldrich) for 1h at room temperature (RT). After one wash in TBST, endogenous peroxidase activity was quenched by incubation with BLOXALL® (Vector Laboratories) for 10 min at RT followed by 2x5 min washes in TBST. Tissues were incubated with the following primary antibodies: PHOX2B (abcam, clone EPR14423, catalog number ab183741, 1:1000), MYCN (Cell Signaling Technology, clone D4B2Y, catalog number 51705, 1:500), CD45 (Cell Signaling Technology, clone D3F8Q, catalog number 70257, 1:1000), CD3(Dako/Agilent, polyclonal, catalog number A0452, 1:2000) diluted in TBST+5% goat serum, in a humid chamber overnight at 4°C. Following 3 x 5min washes in TBST, the slides were incubated in ImmPRESS® HRP Goat Anti-Rabbit IgG Polymer Detection Kit (Vector Laboratories) for 30 min at RT. This was followed by 3 x 5min washes in TBST. For visualization, slides were incubated with ImmPACT® DAB Substrate (Vector Laboratories) for 3-5 min. The sections were counterstained with hematoxylin (abcam) followed by dehydration with graded alcohols and Neo-Clear®. Slides were mounted using SignalStain® Mounting Medium (Cell Signaling Technology). Sections were

scanned at 40x using the Axio Scan.Z1 Digital Slide Scanner, ZEISS. Image analysis was performed using QuPath-0.2.3 [1].

ChIP library preparation and sequencing

Tissue for ChIP sequencing was snap frozen and stored in liquid nitrogen ChIP experiments and library preparation was performed by Active Motif using the HistonePath workflow. Mouse tumor tissues were fixed with 1% formaldehyde for 15 min and quenched with 0.125 M glycine. Chromatin was then isolated by adding lysis buffer followed by tissue disruption with a Dounce homogenizer. Lysates were sonicated and DNA sheared to an average length of 300-500 bp. Genomic DNA (input) was prepared by treating aliquots of chromatin with RNase, proteinase K and heat for de-crosslinking, followed by ethanol precipitation. DNA was quantified on a NanoDrop spectrophotometer. 30 μg chromatin was precleared with protein A agarose beads (Invitrogen) and genomic DNA regions of interest isolated using 4 μg of H3K27Ac antibody (Active Motif, polyclonal, catalog number 39133). Complexes were washed, eluted with SDS buffer, and subjected to RNase and proteinase K treatment. Crosslinks were reversed by incubation overnight at 65 °C, and ChIP DNA was purified by phenol-chloroform extraction and ethanol precipitation. Quantitative PCR reactions were carried out in triplicates on specific genomic regions using SYBR Green Supermix (Bio-Rad). Illumina sequencing libraries were prepared from ChIP and input DNA by end-polishing, dA-addition and adaptor ligation. After PCR amplification, DNA libraries were sequenced on the NextSeq 500 platform (Illumina), 75 bp reads, single-end.

1. Bankhead P, Loughrey M, Fernández J, et al. QuPath: Open source software for digital pathology image analysis. *Sci Rep*. 2017;7(1).

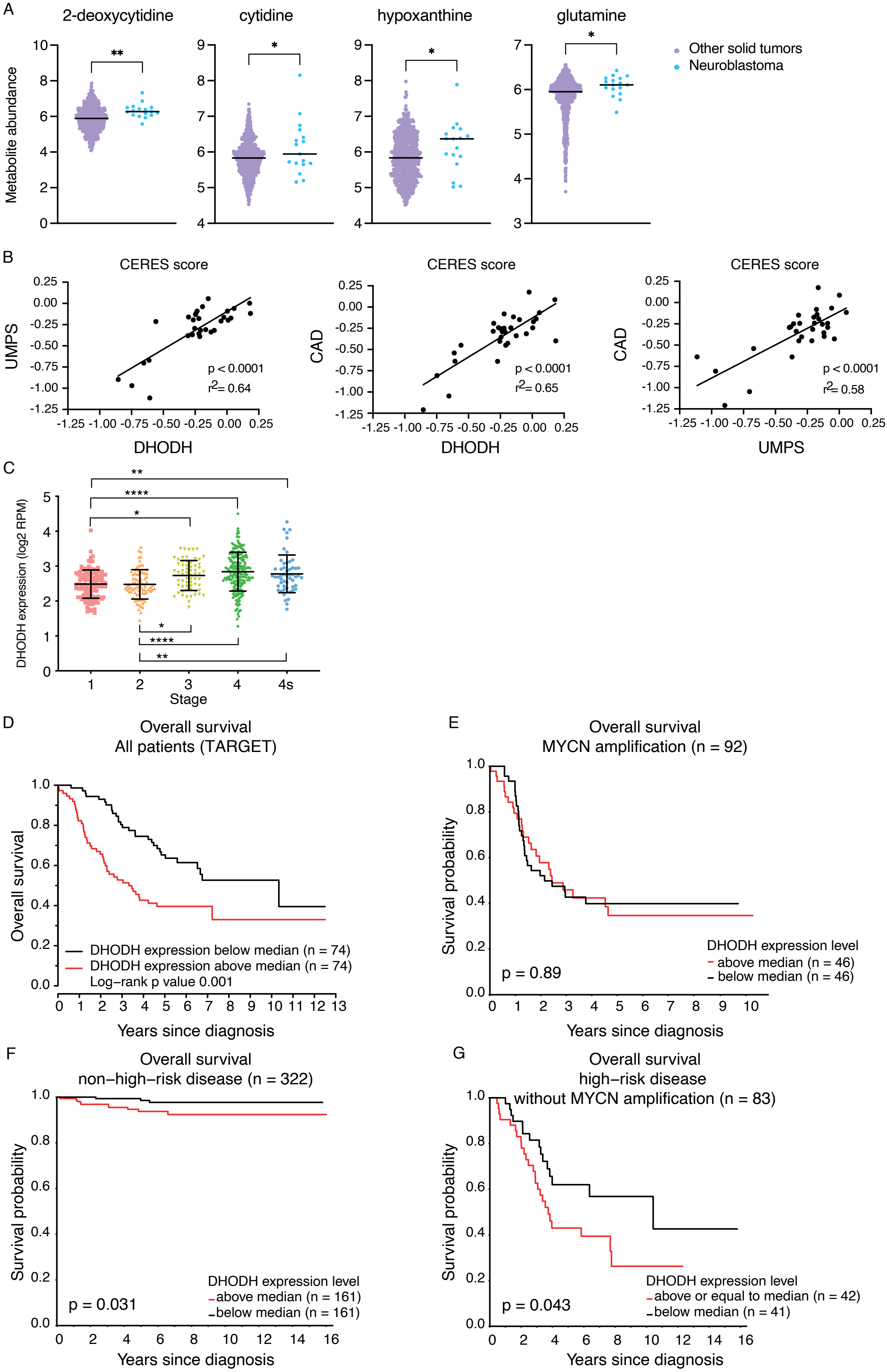
A
2-deoxycytidine
cytidine
hypoxanthine
glutamine
Metabolite abundance
Other solid tumors
Neuroblastoma
B
CERES score
UMPS
DHODH
p < 0.0001
r2= 0.64
CAD
DHODH
p < 0.0001
r2= 0.65
CAD
UMPS
p < 0.0001
r2= 0.58
C
DHODH expression (log2 RPM)
Stage
1
2
3
4
4s
D
Overall survival
All patients (TARGET)
Overall survival
DHODH expression below median (n = 74)
DHODH expression above median (n = 74)
Log−rank p value 0.001
Years since diagnosis
E
Overall survival
MYCN amplification (n = 92)
Survival probability
p = 0.89
DHODH expression level
above median (n = 46)
below median (n = 46)
Years since diagnosis
F
Overall survival
non−high−risk disease (n = 322)
Survival probability
p = 0.031
DHODH expression level
above median (n = 161)
below median (n = 161)
Years since diagnosis
G
Overall survival
high−risk disease
without MYCN amplification (n = 83)
Survival probability
p = 0.043
DHODH expression level
above or equal to median (n = 42)
below median (n = 41)
Years since diagnosis

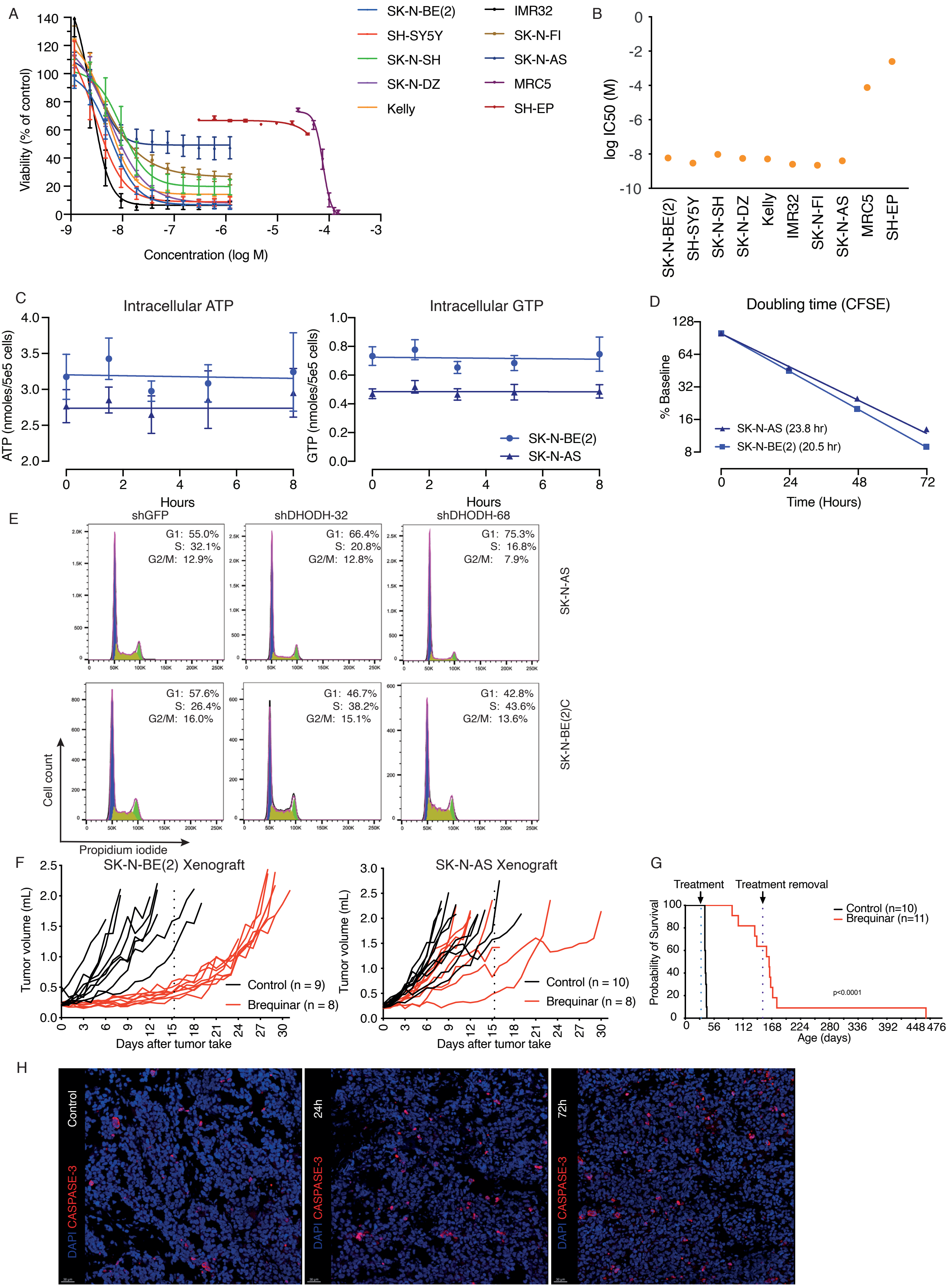

A
Viability (% of control)
Concentration (log M)
SK-N-BE(2)
SH-SY5Y
SK-N-SH
SK-N-DZ
Kelly
IMR32
SK-N-FI
SK-N-AS
MRC5
SH-EP
B
log IC50 (M)
C
Intracellular ATP
ATP (nmoles/5e5 cells)
Hours
Intracellular GTP
GTP (nmoles/5e5 cells)
Hours
SK-N-BE(2)
SK-N-AS
D
Doubling time (CFSE)
% Baseline
SK-N-AS (23.8 hr)
SK-N-BE(2) (20.5 hr)
Time (Hours)
E
shGFP
shDHODH-32
shDHODH-68
G1: 55.0%
S: 32.1%
G2/M: 12.9%
G1: 66.4%
S: 20.8%
G2/M: 12.8%
G1: 75.3%
S: 16.8%
G2/M: 7.9%
SK-N-AS
G1: 57.6%
S: 26.4%
G2/M: 16.0%
G1: 46.7%
S: 38.2%
G2/M: 15.1%
G1: 42.8%
S: 43.6%
G2/M: 13.6%
SK-N-BE(2)C
Cell count
Propidium iodide
F
SK-N-BE(2) Xenograft
Tumor volume (mL)
Control (n = 9)
Brequinar (n = 8)
Days after tumor take
SK-N-AS Xenograft
Tumor volume (mL)
Control (n = 10)
Brequinar (n = 8)
Days after tumor take
G
Treatment
Treatment removal
Probability of Survival
Control (n=10)
Brequinar (n=11)
p<0.0001
Age (days)
H
Control
24h
72h
DAPI CASPASE-3

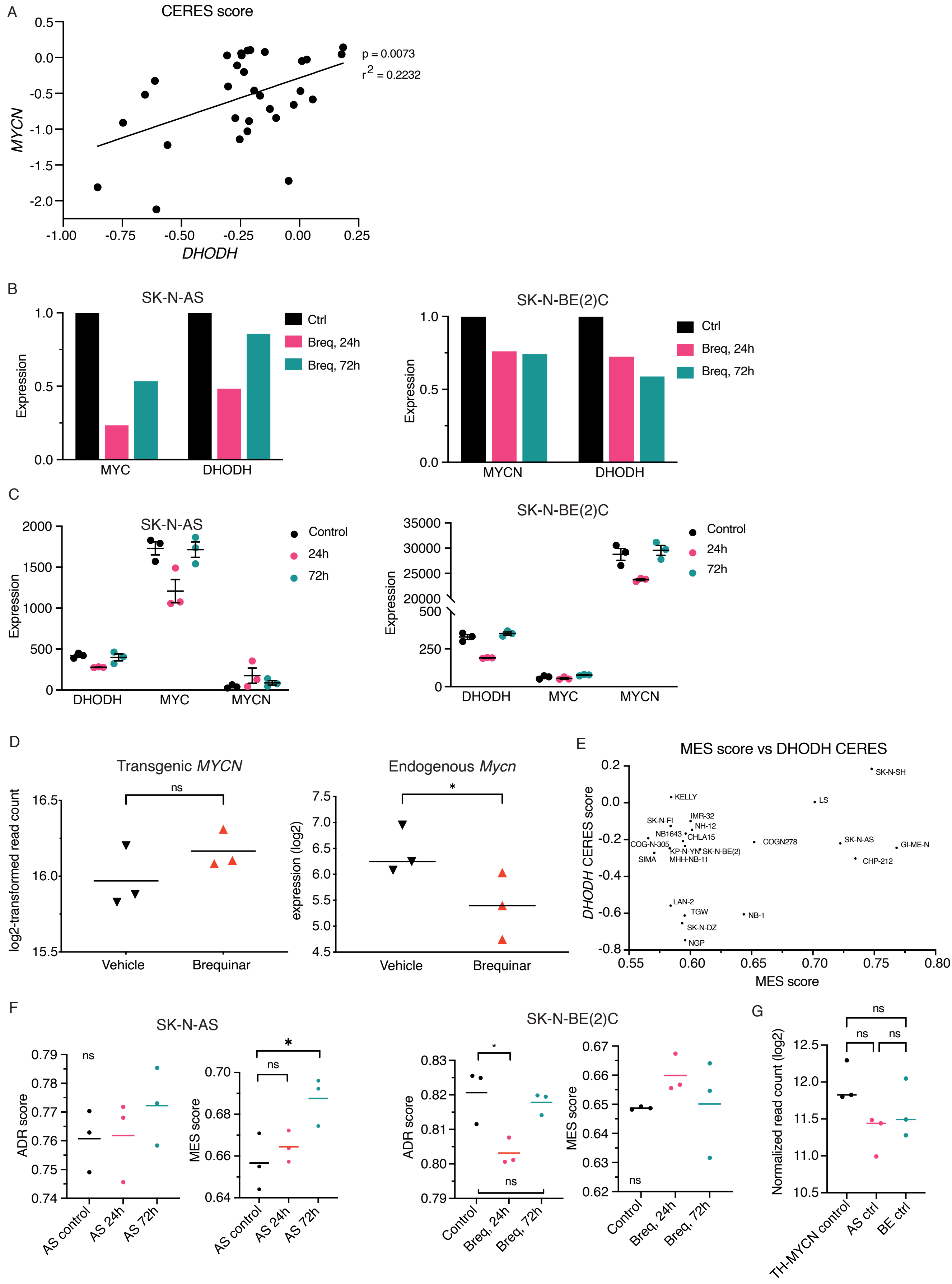

A
CERES score
p = 0.0073
r^2 = 0.2232
MYCN
DHODH
B
SK-N-AS
Ctrl
Breq, 24h
Breq, 72h
Expression
MYC
DHODH
SK-N-BE(2)C
MYCN
C
Control
24h
72h
D
Transgenic MYCN
ns
log2-transformed read count
Vehicle
Brequinar
Endogenous Mycn
*
expression (log2)
E
MES score vs DHODH CERES
DHODH CERES score
MES score
SK-N-SH
KELLY
LS
IMR-32
SK-N-FI
NH-12
NB1643
CHLA15
COG-N-305
COGN278
SK-N-AS
GI-ME-N
SIMA
KP-N-YN
SK-N-BE(2)
MHH-NB-11
CHP-212
LAN-2
TGW
NB-1
SK-N-DZ
NGP
F
ADR score
MES score
AS control
AS 24h
AS 72h
Control
Breq, 24h
Breq, 72h
G
Normalized read count (log2)
TH-MYCN control
AS ctrl
BE ctrl

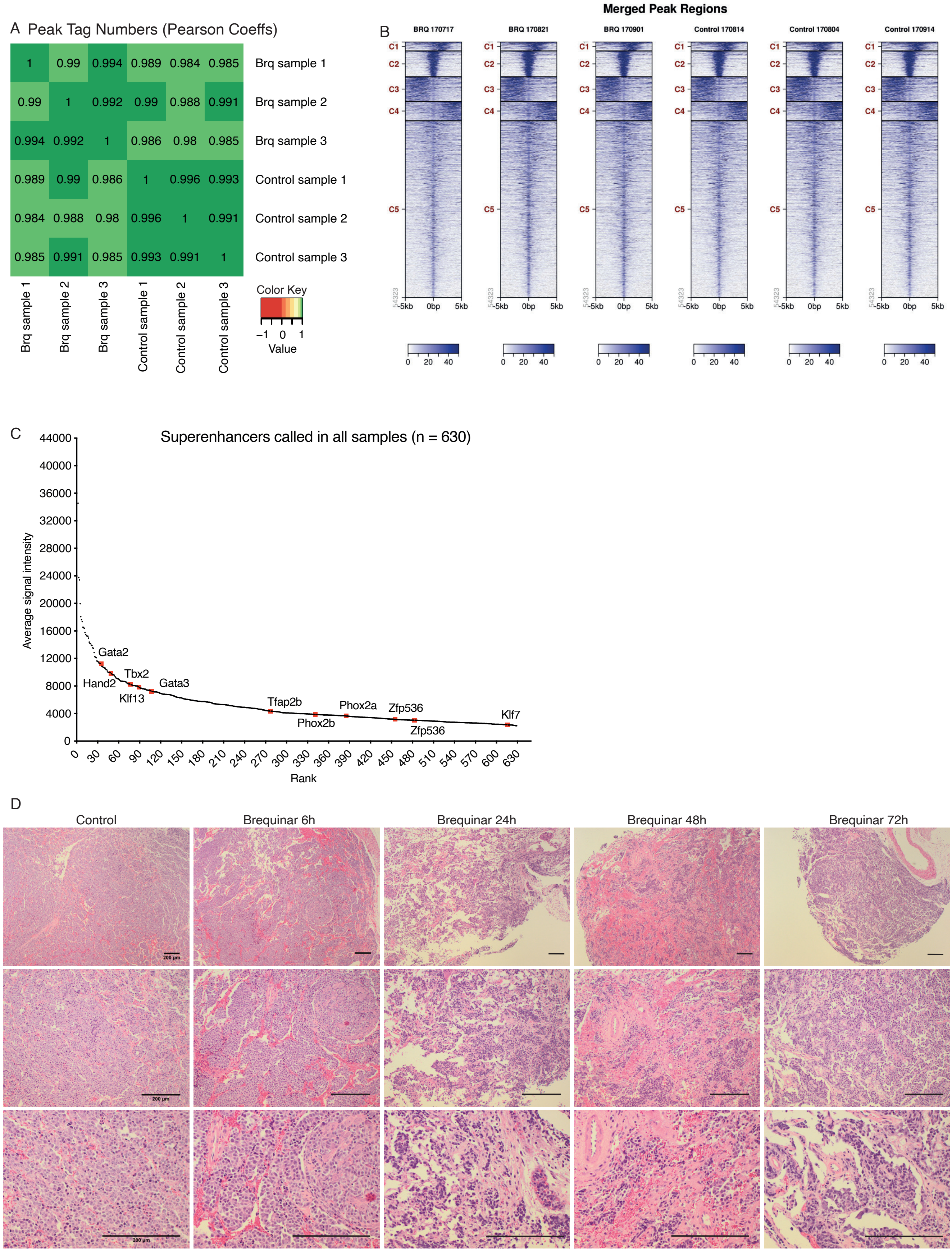
A Peak Tag Numbers (Pearson Coeffs)
1 0.99 0.994 0.989 0.984 0.985 Brq sample 1
0.99 1 0.992 0.99 0.988 0.991 Brq sample 2
0.994 0.992 1 0.986 0.98 0.985 Brq sample 3
0.989 0.99 0.986 1 0.996 0.993 Control sample 1
0.984 0.988 0.98 0.996 1 0.991 Control sample 2
0.985 0.991 0.985 0.993 0.991 1 Control sample 3
Brq sample 1
Brq sample 2
Brq sample 3
Control sample 1
Control sample 2
Control sample 3
Color Key
−1 0 1
Value
Merged Peak Regions
B
BRQ 170717
BRQ 170821
BRQ 170901
Control 170814
Control 170804
Control 170914
C1
C2
C3
C4
C5
54323
-5kb 0bp 5kb
0 20 40
C Superenhancers called in all samples (n = 630)
Average signal intensity
44000 40000 36000 32000 28000 24000 20000 16000 12000 8000 4000 0
Gata2
Hand2
Tbx2
Klf13
Gata3
Tfap2b
Phox2b
Phox2a
Zfp536
Zfp536
Klf7
0 30 60 90 120 150 180 210 240 270 300 330 360 390 420 450 480 510 540 570 600 630
Rank
D
Control
Brequinar 6h
Brequinar 24h
Brequinar 48h
Brequinar 72h
4X
10X
20X
200 µm

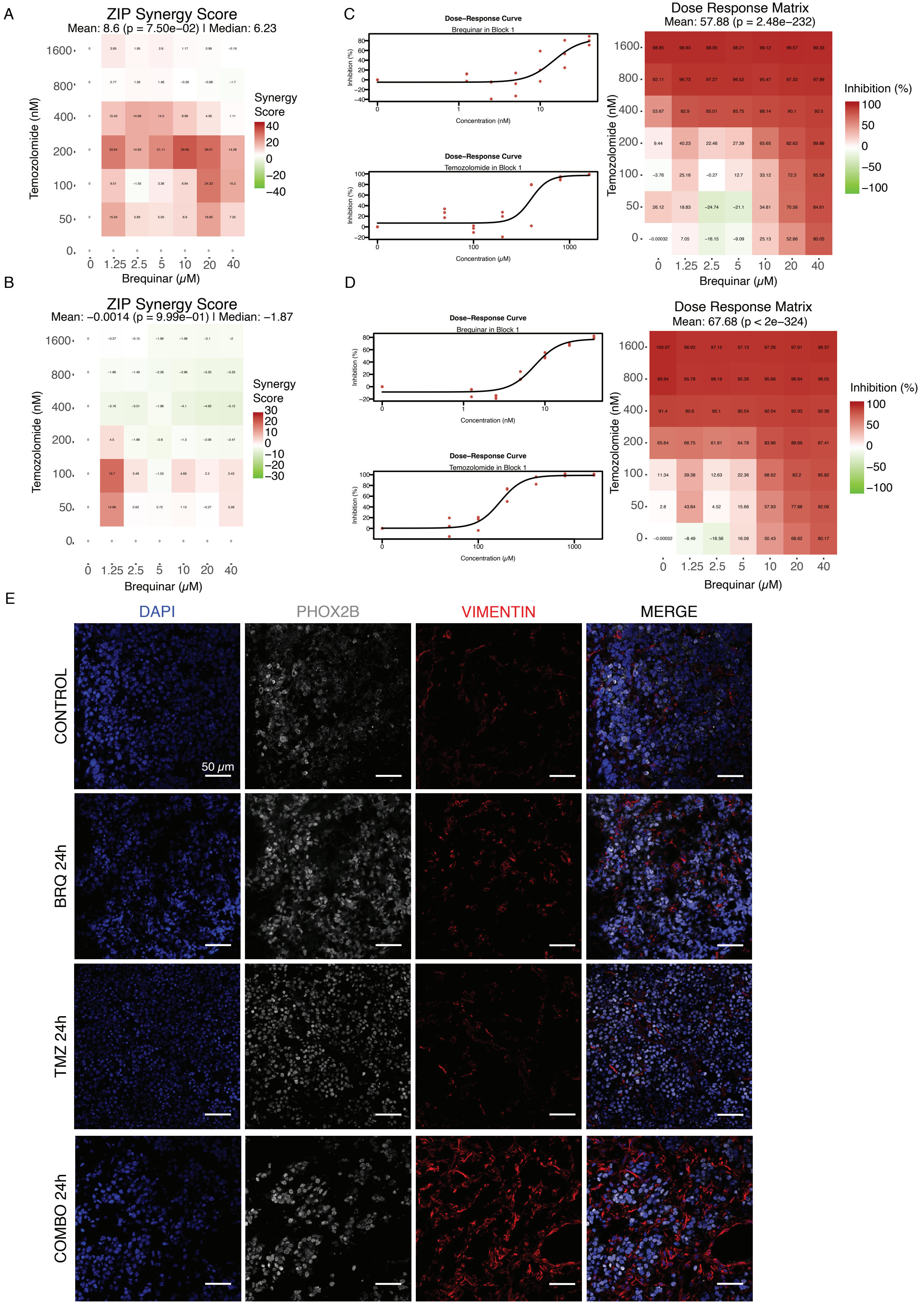
A
ZIP Synergy Score
Mean: 8.6 (p = 7.50e−02) | Median: 6.23
Temozolomide (nM)
Brequinar (μM)
Synergy Score
B
ZIP Synergy Score
Mean: −0.0014 (p = 9.99e−01) | Median: −1.87
C
Dose−Response Curve
Brequinar in Block 1
Temozolomide in Block 1
Inhibition (%)
Concentration (nM)
Concentration (μM)
Dose Response Matrix
Mean: 57.88 (p = 2.48e−232)
Inhibition (%)
D
Dose Response Matrix
Mean: 67.68 (p < 2e−324)
E
DAPI
PHOX2B
VIMENTIN
MERGE
CONTROL
BRQ 24h
TMZ 24h
COMBO 24h
50 μm

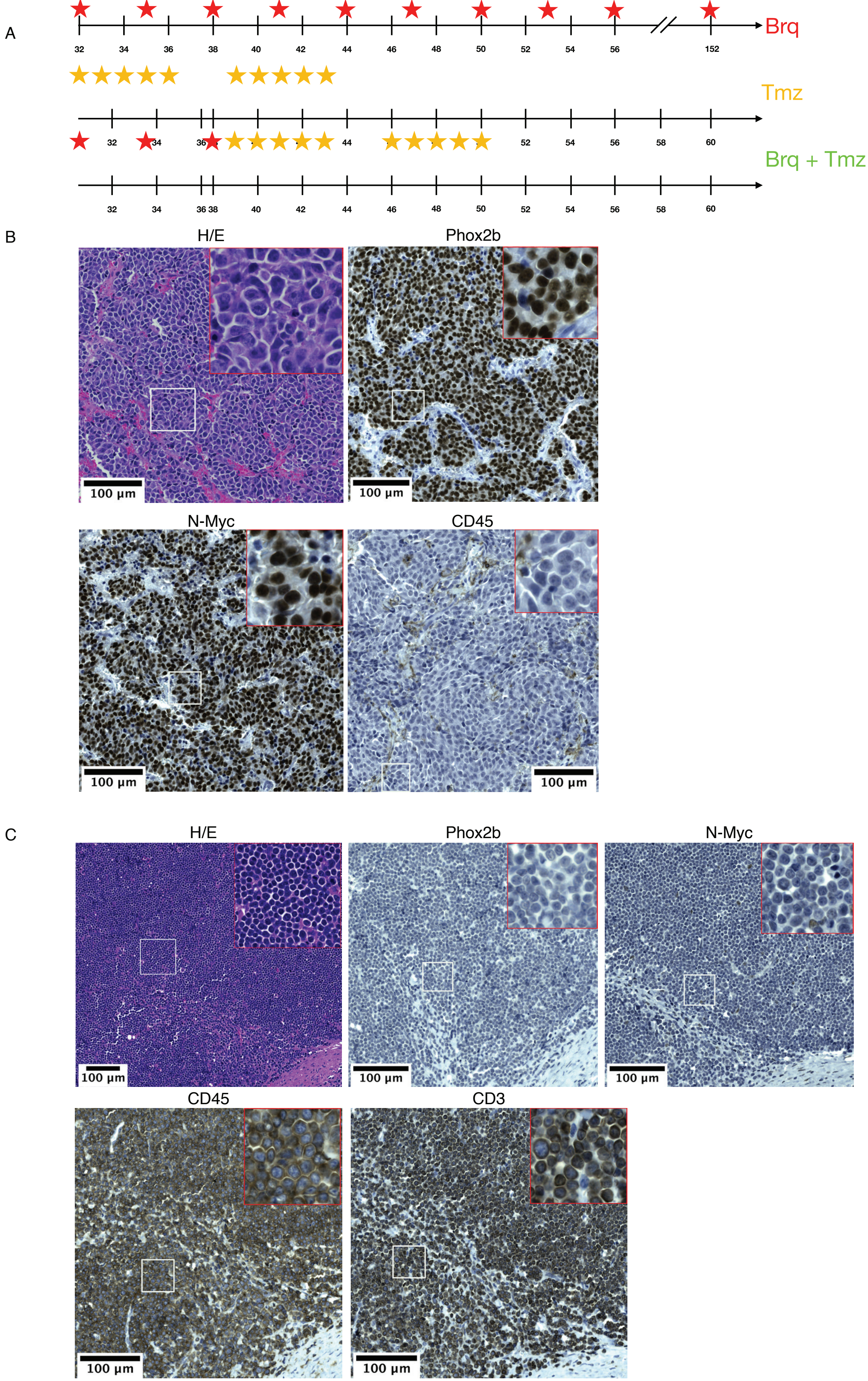
A
Brq
32
34
36
38
40
42
44
46
48
50
52
54
56
152
Tmz
Brq + Tmz
32
34
36 38
40
42
44
46
48
50
52
54
56
58
60
B
H/E
Phox2b
N-Myc
CD45
100 µm
C
H/E
Phox2b
N-Myc
CD45
CD3
100 µm

A

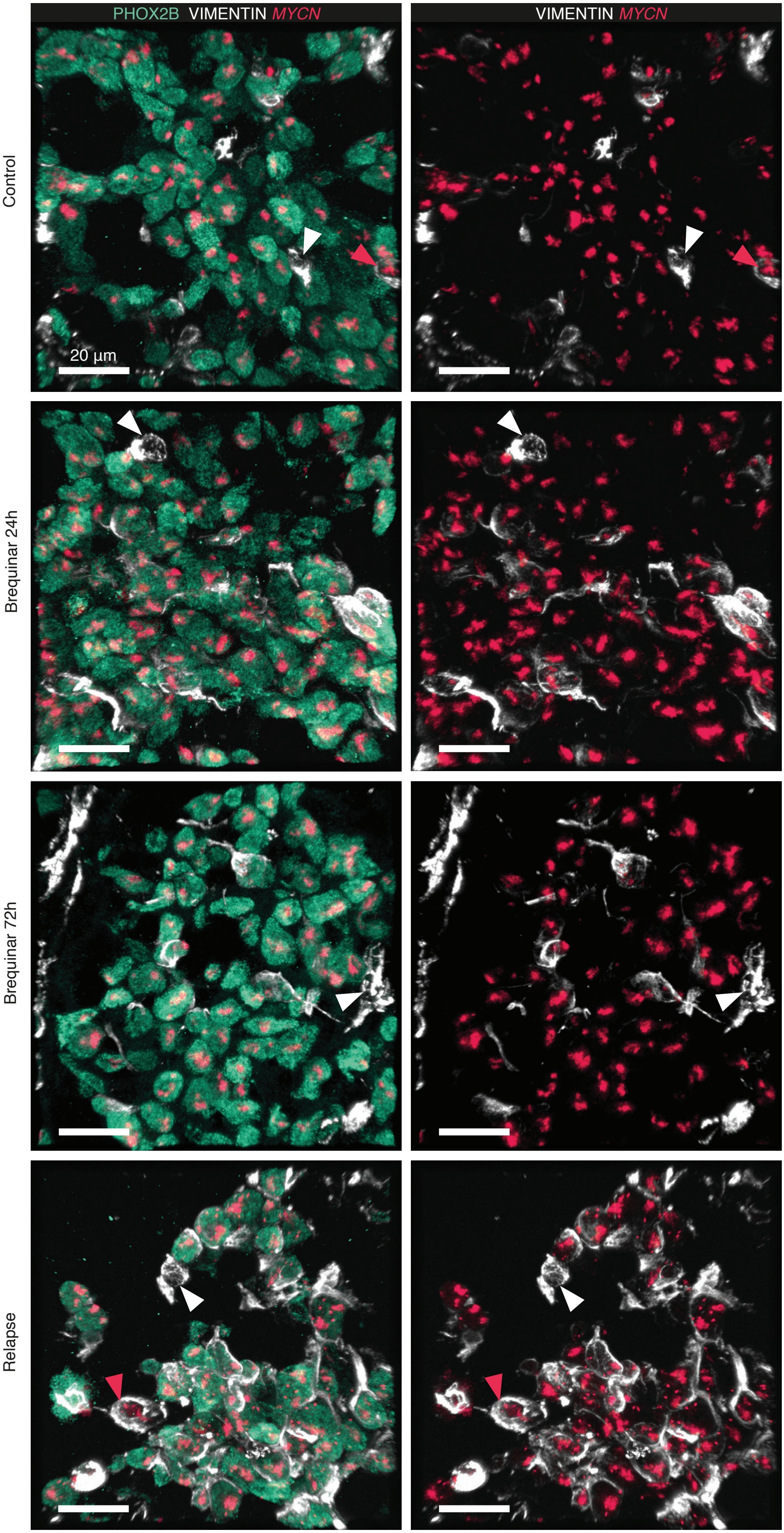
PHOX2B VIMENTIN MYCN
VIMENTIN MYCN
Control
Brequinar 24h
Brequinar 72h
Relapse
20 µm

Supplemental Figure 1. DHODH is highly expressed in neuroblastoma and related to stage, prognosis, and survival. (**A**) Concentrations of nucleotide metabolites significantly accumulated in neuroblastoma compared to other solid tumor cell lines (CCLE metabolomics dataset). Groups are compared using two-tailed t-test. *: $p < 0.05$; **: $p < 0.01$. (**B**) Correlation between UMPS, CAD, and DHODH dependency in neuroblastoma cell lines, DepMap/CCLE dataset. Correlation analyses are performed using simple non-linear regression. Dependency on the three essential enzymes of de novo pyrimidine biosynthesis is highly inter-correlated. (**C**) DHODH expression in SEQC-498 dataset, separated by INSS stage. Groups are compared using one-way ANOVA with multiple comparisons. *: $p < 0.05$, **: $p < 0.01$, ****: $p < 0.0001$. Lines and error bars indicate mean with SD. (**D**) Kaplan-Meier curve of overall survival in high DHODH vs low DHODH group (separated by median), TARGET dataset. Groups are compared using log-rank test. (**E-G**) Kaplan-Meier curves corresponding to the groups in Main Fig 1E showing overall survival separated by DHODH levels above (red) or below (black) median. High DHODH levels are associated with significantly poorer prognosis in the high-risk, non-MYCN amplified and in the non-high risk groups, but not in the MYCN amplified group. Groups are compared using log-rank test.

Supplemental figure 2. DHODH inhibition reduces neuroblastoma growth in vitro and in vivo. (**A**) Dose-response curves of WST-1 assays in brequinar-treated cell lines (72h timepoint). All experiments are performed in independent triplicates. (**B**) log IC50 values calculated from non-linear regression of the data shown in (A). (**C**) Inhibition of DHODH does not affect the pool of purines in neuroblastoma cell lines. Quantification of ATP (left panel) and GTP (right panel) levels in SK-N-BE(2) and SK-N-AS cells following treatment with brequinar (1 μM). (**D**) CFSE assay demonstrating highly similar doubling time in SK-N-AS and SK-N-BE(2) cells. (**E**) Cell cycle analysis by flow cytometry in SK-N-AS and SK-N-BE(2)C cells subject to shRNA knockdown of *DHODH.* DHODH knockdown induces G1 phase arrest in SK-N-AS and S phase arrest in SK-N-BE(2)C. (**F**) Tumor volumes of SK-N-BE(2) and SK-N-AS xenograft tumors in mice treated with brequinar (red). Dotted line

indicates cessation of treatment. Each line represents one mouse. (**G**) Survival of brequinar treated mice after treatment termination (marked with a second dotted line) from the same cohort as shown in Fig. 2E. Groups are compared with log-rank test.

Supplemental Figure 3. *MYCN*-driven neuroblastoma cells are sensitive to DHODH inhibition whereas mesenchymal neuroblastoma cells are less sensitive. (**A**) Scatter plot demonstrating correlation between *MYCN* and *DHODH* gene dependency in neuroblastoma cell lines (DepMap/CCLE dataset). Correlation is analyzed using simple linear regression. (**B**) Expression of *DHODH, MYC,* and *MYCN* genes in SK-N-AS and SK-N-BE(2) cell lines treated with brequinar for 72 hours, measured by qPCR. Expression levels are shown relative to untreated controls. (**C**) Expression of *DHODH, MYC* and *MYCN* in SK-N-AS and SK-N- BE(2) mouse xenograft tumors 24 and 72 hours after one dose of brequinar. Expression levels are shown as normalized read counts (RNA-seq). For both cell lines, there is a transient drop in *DHODH* expression at 24 hours. In SK-N-BE(2), there is a transient drop in *MYCN* expression at 24 hours, whereas there is a transient *MYC* drop in SK-N-AS xenografts at 24 hours. Lines and bars indicate mean and SEM. Groups are compared with one-way ANOVA for multiple groups. (**D**) Expression of human (transgene) *MYCN* and endogenous murine *Mycn* in TH-MYCN tumors 72 hours after one dose of brequinar, measured by RNA-seq. Lines and bars indicate mean values with SEM. ns: not significant; *: $p < 0.05$. Groups are compared by two-tailed t test. (**E**) MES gene expression scores and *DHODH* gene dependency scores (CERES) of neuroblastoma cell lines in the CCLE/DepMap dataset. The highest MES-scoring cell lines all display relatively high CERES scores whereas the most *DHODH*-dependent cell lines all display low MES scores: mesenchymal NB cell lines are less dependent on *DHODH.* (**F**) Scatter plots of ADRN and MES scores in SK-N-BE(2) and SK-N-AS xenografts sampled 24 and 72 hours after one dose of brequinar (50 mg/kg i.p). In SK-N-BE(2), there is a significant, but transient decrease in ADRN score at 24 hours. In SK-N-AS, the MES score is significantly higher at 72 hours.

Groups are compared using one-way ANOVA test with multiple comparisons. *: $p < 0.05$; ns: not significant.

Supplemental figure 4. Transgenic TH-*MYCN* mouse tumors maintain an adrenergic phenotype after brequinar treatment. (**A**) ChIP-seq data of ThMYCN tumors 72 hours after one dose of brequinar. Heatmap showing Pearson correlations between H3K27Ac peak distributions in all sample-wise comparisons. Although the greatest similarities (darker color) are seen within replicates, overall, all samples are highly similar with coefficients > 0,98. (**B**) Heatmaps showing 5 clusters (C1-C5) of merged peak regions across all samples. Color indicates intensity of H3K27 signal (blue = higher signal). Each blue line along the y axis identifies one particular genomic peak region. X axis indicates the distribution of signal across the region: 0 = the center of the region. (**C**) Superenhancer ranking of 630 SEs detected in all six samples (three controls and three brequinar-treated). Core adrenergic transcription factors are highlighted. (**D**) Hematoxylin-eosin (HE) immunohistochemistry stainings of TH-MYCN mouse tumors after one dose of brequinar treatment at various timepoints. The amount of stroma relative to tumor cells increases after brequinar treatment, in particular 48 hours after treatment. Scale bars are indicated in the images.

Supplemental figure 5. Temozolomide and brequinar display synergistic effects in vitro and in vivo*.* (**A-B**) *In vitro* synergy plots from WST-1 cell viability assays in SK-N-BE(2) (panel A) and SK-N-AS(2) (panel B) cells treated with a combination of temozolomide and brequinar. Each cell of the heatmap represents one value in the combination matrix. ZIP scores, quantified by SynergyFinder analysis, are based on three independent replicate experiments. 3D representation of the same data is shown in fig. 4A-B. Brequinar concentrations are shown on the x axis; temozolomide concentrations on the y axis. Red color intensity indicates synergistic effect. (**C-D**) Dose-response curves and heatmaps from

the brequinar/temozolomide *in vitro* drug combination experiment. SK-N-BE(2): panel C, SK-N-AS: panel D. (**E**) Immunostainings (40X) of vimentin (red) and PHOX2B (white) in tumors from TH-MYCN mice treated with one dose of brequinar and/or temozolomide, sampled 24 hours after the last dose. Nuclei were stained with DAPI (blue). Scale bars indicate 50 um.

Supplemental Figure 6.

(**A**) Schematic overview of brequinar (Brq) and temozolomide (Tmz) treatment regimen in transgenic TH-*MYCN* mice. Days after birth is shown on the x axis. Stars indicate treatment days. Brequinar dose: 50 mg/kg i.p; temozolomide dose: 20 mg/kg i.p. (**B-C**) Immunohistochemistry staining of two thoracic tumors seen in TH-*MYCN* mice treated with three doses of brequinar followed by two courses of temozolomide. (**B**): Thoracic lymphoma negative for PHOX2B and MYCN, strongly positive for CD45 and CD3. (**C**): Thoracic neuroblastoma strongly positive for PHOX2B and MYCN with some scattered CD45+ immune cells.

Supplemental Figure 7.

Immunofluorescence images of tumors from brequinar-treated mice bearing SK-N-BE(2)C xenografts: untreated controls, 24h and 72h after one dose of brequinar 50 mg/kg i.p., and relapses after 18 days of brequinar treatment (one dose i.p every three days). Tumors are stained for the ADRN marker PHOX2B (green) and the MES marker vimentin/VIM (white); and FISH for the human *MYCN* locus is performed as a marker of *MYCN*-amplified SK-N-BE(2) cells. ADRN cells are defined as PHOX2B+, VIM-, *MYCN+.* Transitioning cells are defined as PHOX2B+, VIM+, *MYCN+.* Murine stromal cells are defined as PHOX2B-, VIM+, *MYCN-.* White arrow indicates example of murine stromal cell; red arrow indicates example of MES cell. Images are aquired as 63x z-stacks. Scale bar indicates 20 um.

Supplemental Table 1.

Curated list of genes encoding key enzymes of pyrimidine *de novo* synthesis and salvage. For each gene, corresponding gene dependency data in a total of 990 cancer cell lines (DepMap 21Q2 dataset) is provided. For each enzyme that is listed as a dependency in at least one cell line, the top 5 co-dependencies with Pearson correlates are also provided.

Supplemental Table 2.

Results from Cox regression analysis of *DHODH* expression in the SEQC-498 and TARGET datasets.

Supplemental Table 3.

IC50 values and *MYCN* amplification status in a panel of neuroblastoma cell lines.

Supplemental Table 4.

Gene set enrichment analysis (GSEA) of RNA-seq data from brequinar-treated mouse xenografts and TH-MYCN mouse tumors. The MSigDB “Hallmarks” gene sets were used for analysis, providing an overview of relevant biological processes and pathways affected by DHODH inhibition. TSV output files generated by the GSEA software are provided in excel format.

Supplemental Table 5.

Gene set enrichment analysis (GSEA) of RNA-seq data from brequinar treated mouse xenografts and TH-MYCN mouse tumors. The Reactome gene sets were used for analysis and .tsv output files generated by the GSEA software are provided in excel format.

Supplemental Table 6.

Gene set enrichment analysis (GSEA) of RNA-seq data from brequinar treated mouse xenografts and TH-MYCN mouse tumors. The Wikipathways gene sets were used for analysis and .tsv output files generated by GSEA software are provided in excel format.
